# Diversification of LytM Protein Functions in Polar Elongation and Cell Division of *Agrobacterium tumefaciens*

**DOI:** 10.3389/fmicb.2021.729307

**Published:** 2021-08-18

**Authors:** Wanda M. Figueroa-Cuilan, Amelia M. Randich, Caroline M. Dunn, Gustavo Santiago-Collazo, Andrew Yowell, Pamela J. B. Brown

**Affiliations:** ^1^Division of Biological Sciences, University of Missouri, Columbia, MO, United States; ^2^Department of Biology, University of Scranton, Scranton, PA, United States; ^3^Molecular Pathogenesis and Therapeutics Graduate Program, University of Missouri, Columbia, MO, United States

**Keywords:** LytM, bacterial division, divisome, polar growth, DD-endopeptidase, amidase, bacterial polarity, Alphaproteobacteria

## Abstract

LytM-domain containing proteins are LAS peptidases (lysostaphin-type enzymes, D-Ala-D-Ala metallopeptidases, and sonic hedgehog) and are known to play diverse roles throughout the bacterial cell cycle through direct or indirect hydrolysis of the bacterial cell wall. A subset of the LytM factors are catalytically inactive but regulate the activity of other cell wall hydrolases and are classically described as cell separation factors NlpD and EnvC. Here, we explore the function of four LytM factors in the alphaproteobacterial plant pathogen *Agrobacterium tumefaciens*. An LmdC ortholog (Atu1832) and a MepM ortholog (Atu4178) are predicted to be catalytically active. While Atu1832 does not have an obvious function in cell growth or division, Atu4178 is essential for polar growth and likely functions as a space-making endopeptidase that cleaves amide bonds in the peptidoglycan cell wall during elongation. The remaining LytM factors are degenerate EnvC and NlpD orthologs. Absence of these proteins results in striking phenotypes indicative of misregulation of cell division and growth pole establishment. The deletion of an amidase, AmiC, closely phenocopies the deletion of *envC* suggesting that EnvC might regulate AmiC activity. The NlpD ortholog DipM is unprecedently essential for viability and depletion results in the misregulation of early stages of cell division, contrasting with the canonical view of DipM as a cell separation factor. Finally, we make the surprising observation that absence of AmiC relieves the toxicity induced by *dipM* overexpression. Together, these results suggest EnvC and DipM may function as regulatory hubs with multiple partners to promote proper cell division and establishment of polarity.

## Introduction

In bacteria, the peptidoglycan (PG) cell wall plays an essential role in maintaining cell shape, protecting bacteria from environmental stressors, and preventing cell lysis (Scheffers and Pinho, 2005; Vollmer et al., 2008; Cava et al., 2013; Cameron et al., 2015; Ruiz, 2016). The PG cell wall is a net-like structure that consists of glycan strands containing alternating β-1,4-linked *N*-acetylglucosamine (NAG) and *N*-acetylmuramic acid (NAM) sugars crosslinked together through peptide stems (Höltje, 1998). While the bacterial PG cell wall is necessary and sufficient to determine bacterial cell shape, expansion, and separation of the cell wall requires enzymatic action (Uehara and Bernhardt, 2011). In order to expand the existing cell wall, coordination between PG cell wall synthesis and hydrolysis is necessary to allow insertion of new cell wall material at very specific locations within the cell (Vollmer, 2012; Egan et al., 2020).

Bacterial PG hydrolases and autolysins are a highly diverse group of enzymes that contribute to many important processes in the cell, including PG biosynthesis (Singh et al., 2012; Vollmer, 2012; Do et al., 2020), separation of bacterial cells (Heidrich et al., 2001; Uehara et al., 2009, 2010; Goley et al., 2010; Möll et al., 2010, 2014; Yakhnina et al., 2015), peptidoglycan recycling (Jacobs et al., 1995; Langaee et al., 2000; Heidrich et al., 2002; Das et al., 2013; Lamers et al., 2015), and insertion of structures that span the entire cell envelope, including flagella and secretion systems (Scheurwater et al., 2008). During cell division of rod-shaped bacteria, septal PG (sPG) is deposited near mid-cell to build new cell poles prior to cell separation. The final steps of cell division require the coordinated activity of cell wall amidases, endopeptidases, carboxypeptidases, lytic transglycosylases, and regulators of these hydrolyzes called LytM factors to enable cell separation (Heidrich et al., 2001; Peters et al., 2011; Vermassen et al., 2019; Do et al., 2020). In members of the Gammaproteobacteria, including *Escherichia coli*, *Pseudomonas aeruginosa*, and *Vibrio cholerae*, inactivation of amidases prevents cleavage of sPG, leading to cell chaining (Uehara et al., 2010; Möll et al., 2014). Overexpression of amidases results in lysis, suggesting that a tight regulation of PG hydrolases is required to avoid cell lysis. Indeed in *E. coli* and *V. cholerae*, endopeptidases that have lost enzymatic activity are termed “degenerate” LytM (dLytM) factors and function as regulators of amidase activity.

Degenerate LytM factors were first characterized in *E. coli*, where they were reported to have redundant function as late divisome components that assist in the final steps of cell separation (Uehara et al., 2009, 2010). While the single mutants had mild or non-existent phenotypes, the double Δ*envC*Δ*nlpD* mutant exhibited significant defects in cell separation: the mutant formed long chains of regularly spaced cytoplasmic compartments separated by layers of PG and surrounded by a single outer membrane layer (Uehara et al., 2009). Since this early characterization, EnvC*_*Ec*_* and NlpD*_*Ec*_* have been shown to have no endopeptidase activity on their own but instead activate cognate amidases: EnvC*_*Ec*_* specifically activated amidases AmiA and AmiB while NlpD*_*Ec*_* activated AmiC in *in vitro* PG hydrolysis assays (Uehara et al., 2010). In addition, the third *E. coli* dLytM factor, an NlpD paralog (“YgeR” or “ActS”), has been shown to be capable of activating all three amidases with a preference for AmiC (Gurnani Serrano et al., 2021). Structural and mutagenic investigations of EnvC–AmiB interactions have revealed that EnvC uses its deactivated LytM catalytic cleft to bind the autoinhibition helix of AmiB (Peters et al., 2013). The same interface is bound by an autoinhibition alpha helix of EnvC in cocrystals of EnvC and its recruiting partner and regulator FtsX (Yang et al., 2011; Cook et al., 2020). Thus, the dLytM interface of EnvC plays a central role in late divisome regulation. The mechanism of EnvC regulation of amidases is used to explain how other dLytMs regulate amidases, although autoinhibition interactions have not yet been reported for NlpD or YgeR. Recent work in NlpD*_*Ec*_* has indicated that it has additional interactions with the Tol–Pal complex that coordinate peptidoglycan remodeling and outer membrane constriction at the division plane (Tsang et al., 2017). Together, the studies on the *E. coli* dLytM factors suggest that EnvC and NlpD have partially redundant, late division roles in activating cognate amidases to complete the last steps of cell separation as well as unique interactions with other protein networks.

In general, studies of dLytM factors in other proteobacteria have largely supported the evidence from *E. coli* with some striking variation. Although most species have fewer amidases than *E. coli*, at least one amidase has been associated with chaining or cell separation defects: AmiA in *Helicobacter* (Chaput et al., 2016); AmiB in *Vibrio* (Möll et al., 2014) and *Pseudomonas* (Yakhnina et al., 2015); AmiC in *Neisseria* (Garcia and Dillard, 2006; Stohl et al., 2016), *Caulobacter* (Meier et al., 2017; Zielińska et al., 2017; Dubey and Priyadarshini, 2018), and *Hyphomonas* (Cserti et al., 2017); and AmiC1 in *Xanthomonas* (Yang et al., 2018). In most cases, EnvC, NlpD, or both, were implicated in the amidase pathway. For gammaproteobacterium *Xanthomonas campestris* and betaproteobacterium *Neisseria gonorrhoeae*, NlpD has been shown to directly activate AmiC *in vitro* (Stohl et al., 2016; Yang et al., 2018). Both EnvC and NlpD have been suggested to activate AmiB in gammaproteobacteria *V. cholerae* (Möll et al., 2014) and *P. aeruginosa* (Yakhnina et al., 2015) by inference from genetic experiments. In most cases, EnvC and NlpD orthologs were non-essential and resulted in late cell separation defects resulting in a failure to separate after cytokinesis of the inner membrane.

The *E. coli* model for the function of amidases and dLytM factors does not have universal agreement among all proteobacteria, however. Interesting outliers are *Pseudomonas*, which exhibited earlier cell constriction defects for AmiB mutants (Yakhnina et al., 2015) and the pathogens *H. influenzae* (Ercoli et al., 2015) and *N. gonorrhoeae* (Stohl et al., 2016), in which Δ*envC* elicited no morphological or division defects. While EnvC and NlpD have generally been seen as redundant in gamma- and betaproteobacteria, this does not appear to be the case in alphaproteobacteria, where dLytM factors are not strictly conserved, and when they are, their loss has given rise to unique late cell separation phenotypes. Deleting the *envC* ortholog “*lpdF*” in *Caulobacter crescentus* resulted in cells that were mildly chained and electron cryotomography images showed that the cells fail to finish separating PG and OM layers (Zielińska et al., 2017). Loss of the *nlpD* ortholog, “*dipM*,” in *C. crescentus* resulted in a distinct cell separation phenotype that featured completion of inner membrane cytokinesis with deformation and blebbing of the outer membrane at the division plane (Goley et al., 2010; Möll et al., 2010; Poggio et al., 2010). This outer membrane blebbing was similar to Tol–Pal mutants in *Caulobacter* (Yeh et al., 2010) and indicated a relationship between NlpD and the Tol–Pal system much earlier than later determined for *E. coli* (Tsang et al., 2017).

The studies in alphaproteobacteria have presented an additional puzzle. In both *Caulobacter* and *Hyphomonas*, loss of EnvC or AmiC orthologs appeared to produce similar separation phenotypes: mild chaining in *C. crescentus* (Meier et al., 2017; Zielińska et al., 2017) and elongated stalks terminated by chains of bud cells in *Hyphomonas neptunium* (Cserti et al., 2017). These results suggest some sort of regulatory relationship between EnvC and AmiC rather than NlpD and AmiC. While the story is less clear in *H. neptunium*, which only conserves EnvC and not NlpD, various experiments suggest that the two dLyM factors act in at least two, if not three, distinct pathways to drive the final steps of cell separation in *Caulobacter* (Meier et al., 2017; Zielińska et al., 2017). It is possible that this is also true in other proteobacteria but the genetic associations are occluded by redundancy or epistasis, such as in the case of NlpD and the Tol–Pal system in gammaproteobacteria. Accumulating data in various genera supports the assertion that these two dLytM factors are components of molecularly distinct but overlapping protein-interaction networks with different recruitment and activation partners that intersect late in division.

The different phenotypes among proteobacteria are due to divergent genetic backgrounds in which the role of the ortholog, its binding partners, or other intersecting pathways have shifted evolutionarily. In this way, comparative genetic studies across genera can reveal either species-specific functions or conserved roles that are harder to detect in genera with higher levels of redundancy. Here, we sought to expand our understanding of bacterial cell division within the Alphaproteobacteria by exploring the functions of LytM factors and amidases in the bacterial plant pathogen, *Agrobacterium tumefaciens*, an emerging model for the study of cell division in a polar-growing bacterium (Figueroa-Cuilan and Brown, 2018). We find that the LytM factors play multiple roles throughout the *A. tumefaciens* cell cycle. While the catalytically active LytM factors did not make obvious contributions to cell division, the dLytM factors both exhibited strong localization at mid-cell and played distinct, yet potentially overlapping, roles during cell division. Deletion or depletion of dLytM factors resulted in phenotypes that were vastly different from similar mutants in other proteobacteria, where cells fail to separate. In this work, we begin to dissect the functions of these proteins and find that the dLytM factors still likely contribute to the regulation of PG hydrolases but have additional derived functions related to the establishment of growth poles following cell division.

## Materials and Methods

### Bioinformatics and Phylogenetics

Sequences of the LytM genes in Supplementary Table 1 were retrieved by pBLAST searches on the Integrated Microbial Genomes and Microbiomes (IMG/M) database (Chen et al., 2017). Multiple alignments were achieved with MUSCLE (Edgar, 2004) and manually adjusted and visualized with Jalview (Waterhouse et al., 2009). Before performing phylogenetic reconstruction, the sequences were truncated to only the conserved LytM domain. The LytM domain from *S. aureus* (GenBank ID: MBH4889575.1) was used in alignments to determine the boundaries of the conserved domain.

Phylogenetic reconstruction was performed using MEGA11 (Kumar et al., 2018; Stecher et al., 2020) to estimate consensus phylogenies and carry out bootstrapping analysis. The LytM gene tree was inferred by using the Maximum Likelihood method and Le Gascuel 2008 model (Le and Gascuel, 2008). A discrete Gamma distribution was used to model evolutionary rate differences among sites [four categories (+*G*, parameter = 1.6678)]. The rate variation model allowed for some sites to be evolutionary invariable [(+*l*), 0.00% sites]. This analysis involved 178 amino acid sequences (Supplementary Table 1). The tree was visualized and formatted using iTol (Letunic and Bork, 2016). Presence and absence of LytM clade members was determined from the tree and tabulated in Supplementary Table 2.

Full length genes were screened for N-terminal secretory signal sequences using the SignalP-5.0 Server (Almagro Armenteros et al., 2019) and for transmembrane segments using TMHMM (Krogh et al., 2001). Additionally, TREND was used to quickly identify conserved protein domains (Gumerov and Zhulin, 2020) and DeepCoil was used to identify coiled-coil domains (Ludwiczak et al., 2019). The results of this analysis are summarized in Supplementary Table 1.

WebLogo 3 (Crooks et al., 2004) was used to plot the amino acid distribution at each position of the LysM domain. To create the alignments for logo generation, LytM sequences from the defined five clades (and the additional subgroup of active NlpD genes) in the tree were simultaneously aligned to the LytM domain from *S. aureus* (GenBank ID: MBH4889575.1) and any insertions causing gaps in the LytM*_*Sa*_* sequence were removed for uniform comparison. This analysis included the 38 MepM, 36 EnvC, 31 LmdC, 27 NlpD, 6 NlpD (with conserved active site HXXXD, HXH), and 21 LpdB LytM domains from the genes listed in Supplementary Table 1.

### Bacterial Strains and Growth Conditions

*Agrobacterium tumefaciens* C58 and derived strains were grown in LB rich medium or ATGN (0.5% glucose) minimal medium (Morton and Fuqua, 2012) at 28°C with shaking. When appropriate, antibiotics were used at the following working concentrations: kanamycin 300 μg/ml and gentamycin 200 μg/ml. When indicated, IPTG was used as an inducer at a concentration of 1 mM. In some cases, cumate was used as an inducer at a concentration of 0.1 mM. *E. coli* DH5α and S17-1 were routinely cultivated on LB agar or liquid LB medium at 37°C with shaking. When needed antibiotics were used at the following concentrations: kanamycin 50 μg/ml and gentamycin 20 μg/ml. All strains used in this study are listed in Supplementary Table 3. Unless otherwise specified, the experiments were conducted in LB.

### Construction of Strains and Plasmids

All strains and plasmids used are listed in Supplementary Table 3 and primers are listed in Supplementary Table 4. To construct expression vectors containing *lmdC-sfgp*, *rgsM-sfgfp*, *dipM-sfgfp*, and *envC-sfgfp*, the corresponding coding sequence was amplified from purified *A. tumefaciens* C58 genomic DNA without a stop codon. The amplicons and plasmids pSRKKM-Plac-*sfgfp*, pSRKKM-Cym-*sfgfp*, pSRKKM-T7-*sfgfp*, pSRKKM-PenvC-*sfgfp*, were digested overnight and ligated overnight at 4°C using NEB T4 DNA ligase. Ligations were transformed into *E. coli* DH5α and purified plasmids were sequenced to verify expected translational fusions. *Atu1832* was amplified from genomic DNA and inserted into pSRKKM-Cym-*sfgfp* linearized with *Nde*I and *Bam*HI using Gibson cloning (NEB) according to manufacturer’s protocols.

For complementation of Δ*envC*, the EnvC native promoter was cloned into the pSRKKM plasmid. Complementation of Δ*amiC* and Δ*amiD* was achieved by constitutively expressing *amiC* and *amiD* under the T7 medium promoter. The T7 medium promoter was introduced into *Eco*RI and *Nde*I sites of pSRKKM using the oligo annealing/ligation method and the T7forwardEcoRINdeI and T7revEcoRINdeI primers. Lastly, *dipM* with a stop codon was ligated into pSRKKM-Ptac-*sfgfp* to allow conditional overexpression of *dipM* from the Ptac promoter in the presence of IPTG.

### Construction of Deletion/Depletion Plasmids and Strains

Vectors for gene deletions by allelic exchange were constructed using recommended methods for *A. tumefaciens* (Morton and Fuqua, 2012). Gene deletions were achieved by allelic exchange and vectors were constructed as previously described (Howell et al., 2019). Briefly, 500 bp fragments upstream and downstream of the gene of interest (*envC*, *amiC*, *ampD*) were amplified using primer pairs P1/P2 and P3/P4. Overlapping PCR was used to the amplicons generated by P1/P2 and P3/P4, using primer pair P1/P4. The amplicon was digested and ligated into pNTPS139. Ligations were transformed into *E. coli* DH5α and purified plasmids were sequenced to verify expected inserts. The deletion plasmids were introduced into *A. tumefaciens* by mating using an *E. coli* S17 conjugation strain to create kanamycin resistant, sucrose sensitive primary exconjugants. Primary exconjugants were grown overnight in media with no selection. Secondary recombinants were screened by patching for sucrose resistance and kanamycin sensitivity. Colony PCR with primers P5/P6 for the respective gene target was used to confirm deletion. PCR products from P5/P6 primer sets were sequenced to further confirm deletions.

For the insertional knock-out of *Atu1832*, nucleotides 1172–1771 of Atu1832 were amplified from genomic DNA and cloned into pMCS-2 (Thanbichler et al., 2007) linearized with EcoR1 using Gibson cloning. Integrants were isolated by antibiotic selection and checked for correct insertion of the plasmid using primers upstream of this sequence and inside the plasmid.

To construct the DipM and RgsM depletion strains, the target gene was amplified, digested and ligated into the pUC18-mini-Tn7T-GM-Ptac and pUC18-mini-Tn7T-GM-Plac, respectively. The mini-Tn7 vector, along with the pTNS3 helper plasmid, were introduced into C58Δ*tetRA*:a-*att*Tn*7* as described previously (Figueroa-Cuilan et al., 2016). Transformants were selected for gentamycin resistance and insertion of the target gene into the a-*att* site was verified by colony PCR using the tet forward and Tn7R109 primers. PCR products were sequenced to confirm insertion of the correct gene. Next, the target genes were deleted from the native locus as described above in the presence of 1 mM IPTG to drive expression of the target gene from the engineered site. Additional deletions were introduced into the DipM depletion strain following the protocol described above with IPTG present during all steps.

### Fluorescence, Phase Contrast, and DIC Microscopy

Exponentially growing cells (OD600 ∼ 0.6) were immobilized on 1.25% LB or ATGN agarose pads as described previously (Howell et al., 2017). Phase contrast, differential interference (DIC) and epifluorescence microscopy was performed with an inverted Nikon Eclipse TiE equipped with a QImaging Rolera Em-C2 1K EMCCD camera and Nikon Elements Imaging Software. For time-lapse imaging, cells were imaged every 5 or 10 min for the duration of the experiment. Time-lapse microscopy is conducted at room temperature (∼23°C).

To construct demographs of LmdC-GFP, RgsM-GFP, EnvC-GFP, or DipM-GFP localization (Figure 2), cells (∼100 to 500) were imaged for each strain. A GFP channel profile was taken along the medial axis for each imaged cell. These medial axis profiles were then aligned by cell length using MicrobeJ software (Ducret et al., 2016).

**FIGURE 1:**
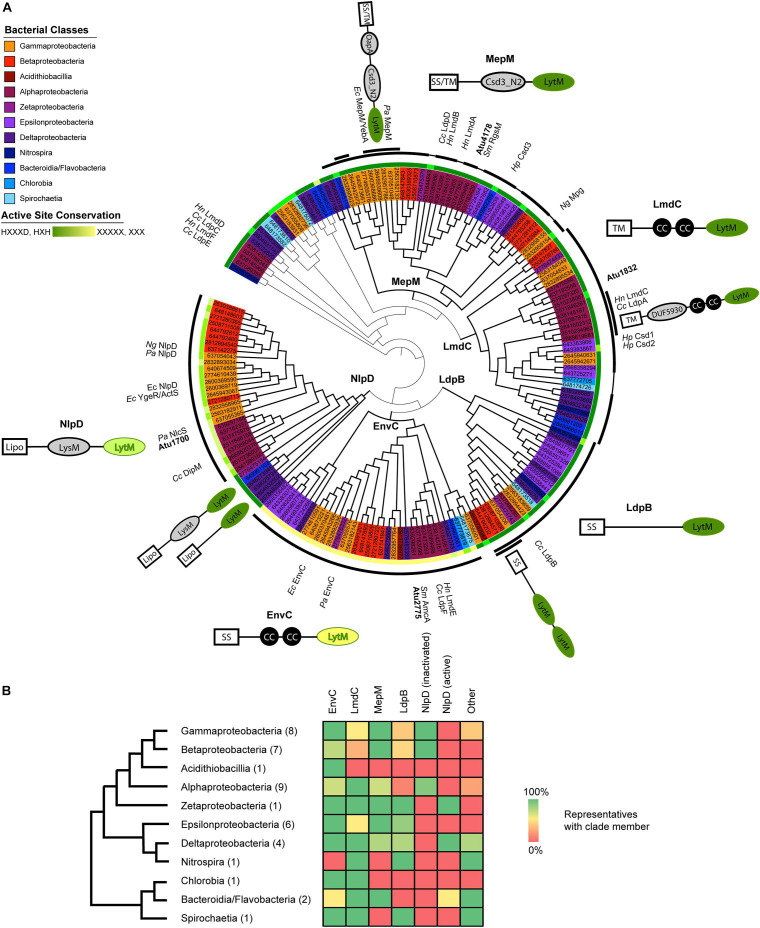
Proteobacterial LytM domain-containing proteins segregate phylogenetically into five major classes with identifiable features outside of the LytM domain. **(A)** Maximum likelihood gene tree of LytM factors constructed with aligned LytM domains from 41 representative species from the Proteobacteria and deep-branching Proteobacteria. The five clades are labeled at their branch point and emphasized by thicker branch weights. This tree has been arbitrarily rooted to clarify the structure of the five clades (see also Supplementary Figure 1). The taxa are colored according to bacterial class (see legend) and shown as JGI ID numbers, which are associated with genetic loci and genomes in Supplementary Table 1. Taxa that have been studied and named are labeled in the outermost ring (*Cc*, *Caulobacter crescentus*; *Ec*, *Escherichia coli*; *Hn*, *Hyphomonas neptunium*; *Hp*, *Helicobacter pylori*; *Ng*, *Neisseria gonorrhoeae*; *Pa*, *Pseudomonas aeruginosa*; *Sm*, *Sinorhizobium meliloti*). The first ring outside of the taxa indicates the degree to which the active site motif (HXXXD, HXH) is conserved, with dark green indicating full conservation and yellow indicating loss of all conservation. See also Supplementary Figure 3 for LytM consensus sequences for each clade. The thick black arcs identify which members of the clade include the schematized N-terminal domains and help distinguish where clades begin and end. Some MepM and LmdC members did not have clade-associated domains, which is indicated by a thinner connecting line within the arc. Some groups of LytM factors shared additional features and these are indicated by a second arc layer. Schematics of characteristic clade architectures appear horizontally next to the clades. LytM domains are colored according to active site conservation. N-terminal domains are colored in gray: LysM, PG-binding domain; CC, coiled-coil motif; DUF5930; Csd3_N2, autoinhibition domain identified in Csd3 of *H. pylori*; OapA, PG-binding domain identified in OapA of *H. influenzae*. Subclade architectures appear at an angle close to the arc of the sequences they represent. Some genes have more copies than the indicated number of N-terminal domains; for signal sequences or domains identified for each LytM factor gene, see Supplementary Table 1. For branch lengths and bootstrap values, see Supplementary Figure 2. **(B)** Presence/absence of LytM clade members in each bacterial class. Bacterial classes are arranged in a cladogram drawn using phylogenies constructed from concatenated gene trees (Wu et al., 2009; Kysela et al., 2016). The number of representatives of each class is shown in parentheses. Presence/absence is indicated in the heat map using a gradient of green (100%) to yellow (50%) to red (0%). Only the genes in the tree in **(A)** are included. See Supplementary Table 2 for presence/absence data for each species.

**FIGURE 2:**
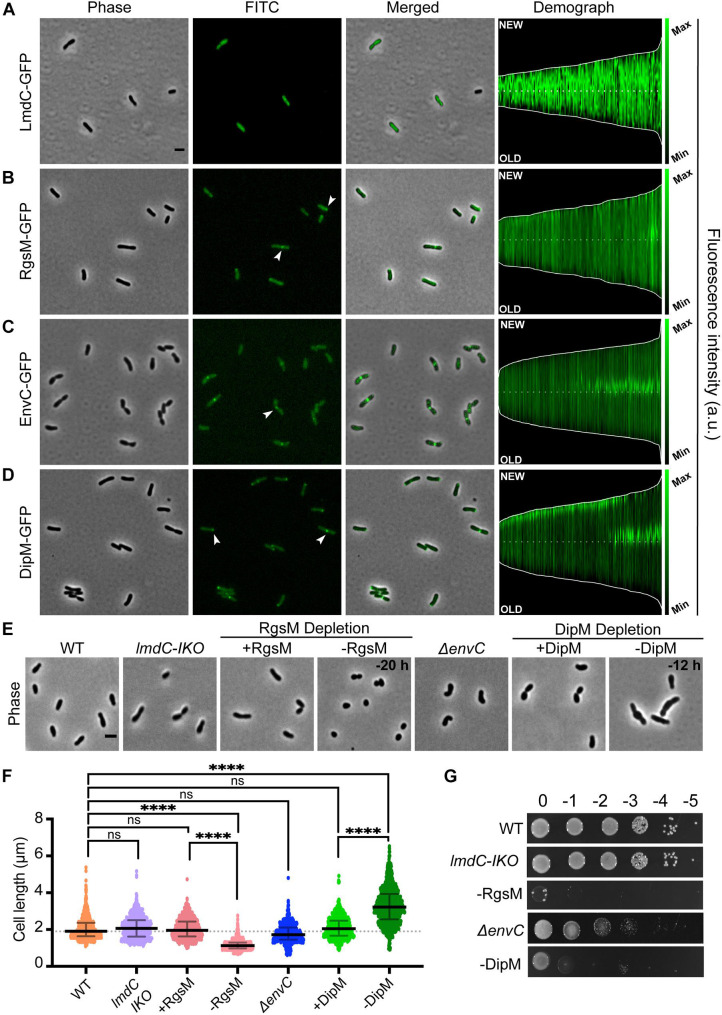
*Agrobacterium tumefaciens* encodes four of the major LytM classes, each with distinct localization patterns and functional roles. (**A–D**, left) Subcellular localization patterns for each LytM-domain containing factor in wildtype (WT) cells. Cells expressing either LmdC-GFP (Pcym-LmdC), RgsM-GFP (Pcym-RgsM), EnvC–GFP (PenvC-EnvC), or DipM-GFP (PT7-DipM) were grown to exponential phase (OD600 ∼ 0.6) and imaged by phase and fluorescence microscopy. Scale bar = 2 μm. (**A–D**, right) Demographs depict the localization of each LytM-domain containing factor at the population level. Median profiles of GFP channel of ∼100 to 500 cells per strain were stacked and ordered by cell length. “New” = active growth pole, “Old” = inert pole. **(E)** Phase contrast microscopy of representative strains. **(F)** Cell length distribution of indicated strains grown in liquid media. The cell length distribution is shown in scattered dot plots. The middle dash line represents the WT median cell length, the error bars represent the interquartile range of the population. ****P < 0.0001, ns, not significantly different. **(G)** Cell viability for each strain is shown by spotting serial dilutions. IKO indicates an insertional knockout strain. All data collected for this figure was obtained from cells grown in LB.

### Quantitative Image Analysis of Cell Growth and Morphology

Exponentially growing cells were imaged using phase microscopy as described above. Bacterial cell length was detected by MicrobeJ software whereas principal component analysis (PCA) to identify shape variations was performed using CellTool (Pincus and Theriot, 2007). Phase contrast images were converted and edited to binary masks in FIJI (Schindelin et al., 2012) before using CellTool.

### Cell Viability Assays

For cell viability spot assays, cultures were grown overnight and diluted to an OD600 = 0.05 and serially diluted in LB or ATGN agar plates. Four microliters of each dilution was spotted and plates were incubated at 28°C for 36 h (LB) or 48 h (ATGN) before imaging. When appropriate plates contained 300 μg/ml kanamycin and/or IPTG at 1 mM as indicated in figure legends. For the RgsM, DipM, or DipM-derived depletion strains, cells were grown in liquid media in the presence of 1 mM IPTG to an OD600 = 0.6. The cells were washed three times by centrifugation in media to remove the inducer and resuspended to an OD600 = 0.05 in media. Serial dilutions were performed, and 4 μl of each dilution was spotted onto and plates were incubated at 28°C for 36 or 48 h before imaging. Each condition was tested in duplicate during three independent experiments (*n* = 3).

### Outer Membrane Integrity Assay

For the SDS susceptibility assay, exponentially growing cells were diluted to an OD600 = 0.5. A total of 0.25 ml of each culture was aliquoted into 1.5 ml tubes and centrifuged at 4.5 × *g* for 5 min. Cells were resuspended in 0.25 ml of various concentrations (0.625, 1.25, 2.5, and 5%) of SDS solubilized in HEPES buffer at a pH 7.4, and incubated for 5 min. Untreated controls were resuspended in 0.25 ml of HEPES buffer only. Four microliter of each dilution was spotted onto LB and stored at 28°C for 24 h before imaging. When treating depletion strains with SDS, cells were pre-depleted as indicated, treated with SDS for 5 min and spotted on plates containing IPTG and no SDS. To determine the strain sensitivity to high salt, cell viability assays were conducted as described above on LB plates containing 2% NaCl.

### Whole Cell TEM, SEM, and Thin Section TEM

Wildtype and Δ*envC* strains were grown in LB or ATGN for ∼16 h to early stationary or late exponential, respectively, before being spun down and resuspended in fixative (2% paraformaldehyde, 2% glutaraldehyde in 100 mM sodium cacodylate buffer pH = 7.35). DipM depletion strains were grown as described and depleted for the indicated time before being collected, spun down, and resuspended in fixative. Unless otherwise stated, all reagents were purchased from Electron Microscopy Sciences and all specimen preparation was performed at the Electron Microscopy Core Facility, University of Missouri.

For whole cell TEM, fixed whole cells were rinsed by pelleting at 2,500 g and resuspending in water. Samples were placed on negatively charged carbon coated copper grids. A negative charge was applied to the grid using a PELCO easiGlow Glow Discharge Cleaning System. Cells were allowed to settle on prepared grids for 2 min before removing the water solution and replacing it with 1% aqueous uranyl acetate for 2 min before drying and imaging. Images were acquired with a JEOL JEM 1400 Transmission Electron Microscope (JEOL, Peabody, MA, United States) at 80 kV on a Gatan Ultrascan 1000 CCD (Gatan, Inc., Pleasanton, CA, United States).

For SEM, fixed whole cells were plated overnight on cell culture treated coverslips to ensure adhesion and rinsed with 100 mM sodium cacodylate buffer, pH 7.35 containing 130 mM sucrose. Secondary fixation was performed using 1% osmium tetroxide (Ted Pella, Inc., Redding, CA, United States) in cacodylate buffer using a PELCO BioWave (Ted Pella, Inc., Redding, CA, United States) operated at 100 W for 1 min. Specimens were next incubated at 4°C for 1 h, then rinsed with cacodylate buffer and further with distilled water. Using the PELCO BioWave, a graded dehydration series (per exchange, 100 W for 40 s) was performed using ethanol. Samples were dried using the Tousimis Autosamdri 815 (Tousimis, Rockville, MD, United States) and samples were sputter coated with 5 nm of platinum using the EMS 150T-ES Sputter Coater. Images were acquired with a FEI Quanta 600F scanning electron microscope (FEI, Hillsboro, OR, United States) at 2 kV, Spot 7, with the Everhart–Thornley secondary electron detector.

For thin sectioning, each sample was centrifuged at 2,500 × *g* and the resulting pellet was resuspended in HistoGel (Thermo Scientific, Kalamazoo, MI, United States). Next, fixed pellets were rinsed with 100 mM sodium cacodylate buffer, pH 7.35 (Sigma Aldrich, St. Louis, MO, United States), and 130 mM sucrose. Secondary fixation was performed using 1% osmium tetroxide (Ted Pella, Inc., Redding, CA, United States) in 2-ME buffer using a PELCO BioWave (Ted Pella, Inc., Redding, California) operated at 100 W for 1 min. Specimens were next incubated at 4°C for 1 h, then rinsed with cacodylate buffer and further with distilled water. En bloc staining was performed using 1% aqueous uranyl acetate and incubated at 4°C overnight, then rinsed with distilled water. Using the PELCO BioWave, a graded dehydration series (per exchange, 100 W for 40 s) was performed using ethanol, transitioned into acetone, and dehydrated specimens were then infiltrated with EPON resin (250 W for 3 min) and polymerized at 60°C overnight. Sections were cut to a thickness of 75 nm using an ultramicrotome (Ultracut UCT, Leica Microsystems, Germany) and a diamond knife (Diatome, Hatfield, PA, United States). Images were acquired with a JEOL JEM 1400 Transmission Electron Microscope (JEOL, Peabody, MA, United States) at 80 kV on a Gatan Ultrascan 1000 CCD (Gatan, Inc., Pleasanton, CA, United States).

### Western Blotting

For monitoring DipM expression in LB and ATGN, 20 ml cultures were grown to an OD600 = 0.6 in the presence of IPTG. A 2-ml aliquot of the culture was removed from each culture, and the pellets were stored to serve as time zero (DipM-replete). The remaining 18 ml of cultures were washed three times with LB or ATGN *via* centrifugation to remove the IPTG. The washed pellets were resuspended and normalized to an OD600 of 0.4 and kept in exponential phase throughout the experiment. Aliquots were collected after 2, 4, 8, 16, and 24 h to monitor the levels of DipM production during the depletion. The cell pellets were stored at -80°C until further analysis. The cell pellets were incubated with 100 μl of a master mix containing 1 ml of BugBuster protein extraction reagent (Novagen) and supplemented with 1 (EDTA-free protease inhibitor cocktail (Sigma), 10 μl of lysonase (Novagen), 2,500 U/ml DNase I (Thermo Scientific), and 1 mM dithiothreitol (DTT) (Thermo Scientific) for 30 min with shaking at room temperature to lyse the cell pellets. The whole-cell lysates were clarified by centrifugation at 17,136 × *g* for 15 min. The BCA protein concentration kit (Thermo Scientific Pierce) was used to measure the total protein concentration of each sample. A final concentration of 1 × Laemmli buffer (BioRad) was added to the cleared cell lysates. Samples were boiled at ∼100°C for 5 min prior to loading equivalent amounts in each well of an SDS-10% PAGE gel. The separated proteins were electroblotted onto polyvinylidene difluoride (PVDF) membranes (Bio-Rad) and blocked for 1 hour in 5% non-fat dry milk powder solubilized in 1% TBST (1 × Tris-buffered saline, 1% Tween 20). The blocked PVDF membranes were probed primary antibody (*Caulobacter crescentus* α-DipM diluted 1:2,000) for 1.5 h in 2.5% milk-TBST, followed by incubation with secondary antibody (horseradish peroxidase-conjugated goat anti-rabbit at a dilution of 1:10,000) for 1 h in 2.5% milk-TBST. The secondary antibody was detected using the ECL Plus-HRP substrate (Thermo Scientific Pierce).

### Quantification of Cell Cycle and Polarity Phenotypes

**FIGURE 3:**
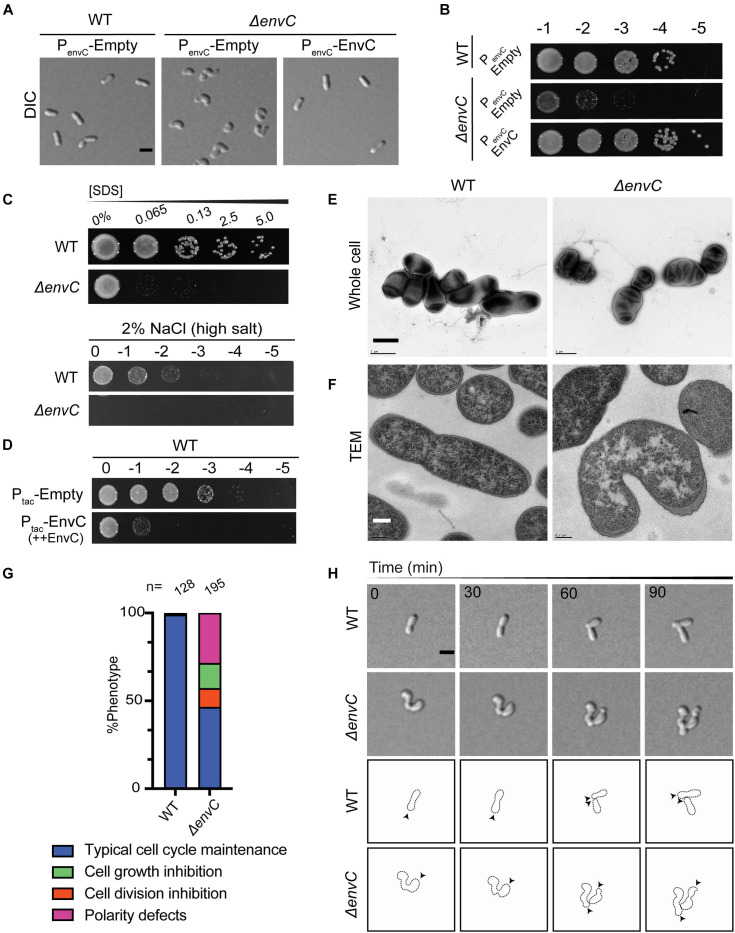
Loss of *envC* results in cell curvature, decreased viability, and OM destabilization in *A. tumefaciens*. **(A)** DIC images of exponentially growing WT cells containing empty plasmid (PenvC-Empty), Δ*envC* containing empty plasmid (PenvC-Empty), or Δ*envC* expressing *envC via* its native promoter [PenvC-EnvC (complementing plasmid)]. **(B)** Cell viability of WT cells expressing empty plasmid (PenvC-Empty), Δ*envC* containing empty plasmid (PenvC-Empty), or Δ*envC* expressing *envC via* its native promoter [PenvC-EnvC (complementing plasmid)] is shown by spotting serial dilutions. **(C)** SDS and salt sensitivity of WT and Δ*envC*. Briefly, for the SDS assay, exponentially growing cells were treated with various concentrations of SDS for 5 min and spotted on LB plates and analyzed 24 h post incubation. To assess salt sensitivity, exponentially growing WT and Δ*envC* were serially diluted and spotted on LB solid medium containing 2% NaCl (high salt) and analyzed ∼36 h post incubation. **(D)** Cell viability of WT strain after overexpression of empty plasmid (Ptac-Empty) or *envC* under the control of the tac promoter (Ptac–EnvC). **(E)** TEM of whole cells of WT (left) *A. tumefaciens* or the Δ*envC* mutant (right). Cells were grown ∼16 h in LB to early stationary phase, fixed, and stained with 2% uranyl acetate. Scale bar = 1 μm. **(F)** Thin section TEM of WT (left) *A. tumefaciens* or the Δ*envC* mutant (right). Cells from **(E)** were embedded in resin and prepared as described in the methods. Scale bar = 0.2 μm. **(G)** Quantitative analysis of the phenotypes of the WT and Δ*envC* strains. % Phenotype was calculated by counting the number of cells in a WT and Δ*envC* time-lapses displaying one of the phenotypes indicated and dividing it by the total number of cells per strain. (**H**, top) Time-lapse microscopy of WT and Δ*envC* over the course of 90 min. Scale bar = 2 μm. (**H**, bottom) Schematic of WT and Δ*envC* time-lapses. Arrows indicate active growth. All data collected for this figure was obtained from cells grown in LB.

**FIGURE 4:**
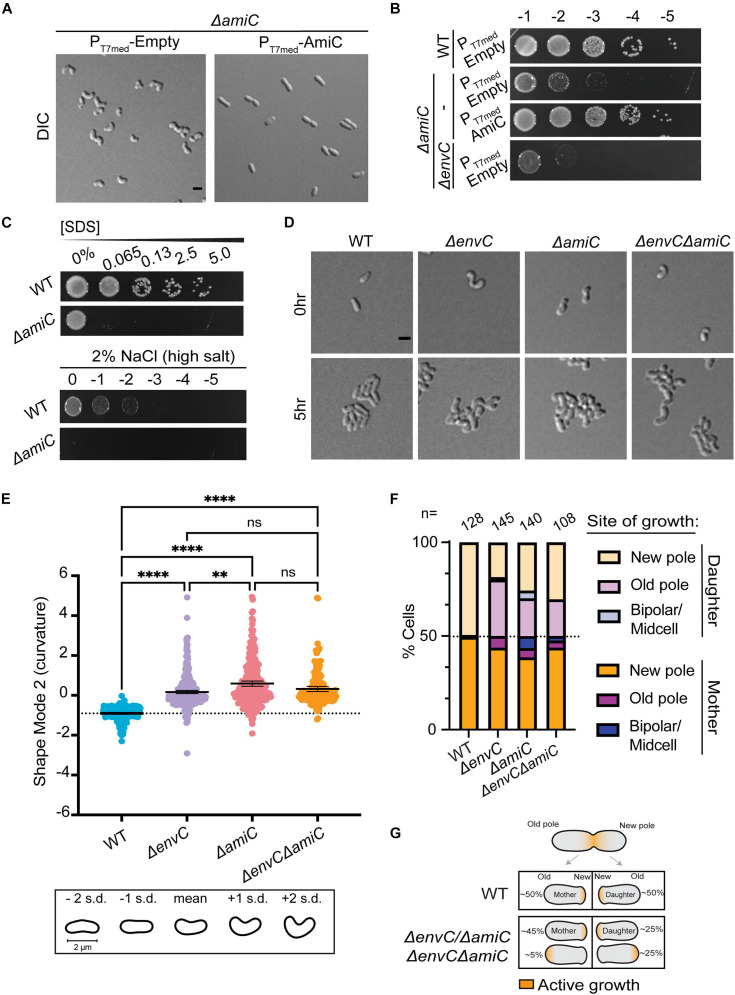
EnvC likely functions in the same pathway as AmiC to help dictate proper polar patterning in *A. tumefaciens*. **(A)** DIC images of exponentially growing WT cells containing empty plasmid (PT7med-Empty), Δ*amiC* containing empty plasmid (PT7med-Empty), or Δ*amiC* constitutively expressing *amiC via* the T7 medium promoter [PT7med-AmiC (complementing plasmid)]. Scale bar = 2 μm. **(B)** Cell viability of WT cells containing empty plasmid (PT7med-Empty), Δ*amiC* containing empty plasmid (PT7med-Empty), or Δ*amiC* constitutively expressing *amiC via* the T7 medium promoter [PT7med-AmiC (complementing plasmid)] is shown by spotting serial dilutions. **(C)** SDS and salt sensitivity of WT and Δ*amiC* as described in Figure 3. **(D)** Cell morphology and microcolony formation are shown for WT, Δ*envC*, Δ*amiC*, and Δ*envC*Δ*amiC* at 0 and 5 h. Scale bar = 2 μm. **(E)** Scatter plot depicting shape mode 2 (curvature) from PCA of cell shape for WT, Δ*envC*, Δ*amiC*, and Δ*envC*Δ*amiC*. **(F)** Quantification of sites of growth initiation following cell division events captured during time-lapse imaging in WT, Δ*envC*, Δ*amiC*, and Δ*envC*Δ*amiC*. Daughter cells are defined as cells that inherit the former growth pole. **(G)** Schematic representation of cell growth polarity inheritance. In WT cells, most mother and daughter cells resume polar elongation at the new pole after completing cell division. However, in the absence of *envC*, *amiC*, or both, about 50% of the daughter cells initiate polar growth at the old pole. All data collected for this figure was obtained from cells grown in LB. **P < 0.01; ****P < 0.0001; ns, not signficant.

For Figure 3G, DIC time-lapse microscopy of cells growing on agarose pads (as described above) was used to monitor cell cycle progression. Typical cell cycle maintenance indicates that an individual cell elongates, constricts at mid-cell, and divides with growth resuming at new poles generated by cell division. Cell growth inhibition indicates that the cell stops growing; cell division inhibition indicates that cells do not properly constrict or divide; and polarity defect indicates that growth appears to occur from an old pole. The % of cells displaying each cell cycle phenotype was calculated by counting the number of cells displaying the indicated phenotypes and dividing it by the total number of cells analyzed for each strain. For Figure 4E, DIC time-lapse microscopy was employed to track growth poles for 1–3 generations. In WT cells, elongation occurs at the new poles generated by cell division in both mother cells and daughter cells. Mother cells are defined as the cells which inherit the oldest (non-growth) pole when a cell divides whereas daughter cells inherit the former growth pole. Cells resuming growth at the newly formed cell poles were categorized as growing through the new pole. Cells resuming growth at the pole opposite to the new pole generated by cell division were categorized as resuming growth at the old pole.

## Results

### The Four LytM Factors of *A. tumefaciens* Exhibit Distinct Localization Patterns and Functions With No Redundancy

The *A. tumefaciens* genome encodes four open reading frames that contain LytM (M23) enzymatic domains. Because of the wide diversity of genes containing LytM domains among Proteobacteria, it can be difficult to infer whether a given factor should have an ancestral or species-specific role. We took a phylogenetic approach to place the four LytM factors within a greater functional and evolutionary context: we collected all detectable LytM factors from representatives of all the proteobacterial classes, including some deep-branching proteobacterial classes, and constructed a maximum likelihood gene tree using only the LytM domains (Figure 1A). In this gene tree, five clades emerged that were distinguished by the conservation of N-terminal protein domains outside of the LytM domain in the full length genes (see also Supplementary Figures 1, 2). All of these clades have characterized members, and we named the clades using the most characterized member. In general, almost all surveyed bacterial representatives encoded orthologs of EnvC, LmdC, and MepM whereas Csd and NlpD orthologs were constrained to or have been lost in certain bacterial classes (Figure 1B). Divisome components NlpD and EnvC were further distinguished from other LytM factors and each other by dLytM domains with distinct inactivation patterns of the catalytic site (Figure 1A and Supplementary Figure 3). LytM endopeptidases belong to the large family of lysostaphin-type metalloenzymes that conserve a zinc chelating active site that catalyzes the cleavage of peptide bonds (Firczuk and Bochtler, 2007). This catalytic site is defined by the conserved signature sequence HXXXD, HXH (Bochtler et al., 2004; Firczuk et al., 2005; Grabowska et al., 2015). Crystal structures of LytM domains with substitutions at these positions do not contain the Zn^2+^ cofactor (Peters et al., 2013; An et al., 2016; Cook et al., 2020). The inactivation of the catalytic site has been well-documented among various studies of dLytM factors in the Proteobacteria and it has been shown in various members that these factors activate cognate amidases rather than directly hydrolyzing peptide bonds themselves (Uehara et al., 2009; Stohl et al., 2016; Yang et al., 2018; Gurnani Serrano et al., 2021).

The *A. tumefaciens* genome encodes four of the five major LytM factor classes detected in our approach. The genome encodes two enzymatically active LytM endopeptidases and two dLytM factors. It does not encode a member of the LpdB clade, which is particularly conserved in the Delta- and Epsilonproteobacteria and Spirochaetia (Figures 1A,B). This clade features no distinguishing domains by sequence analysis and we were unable to determine if any orthologs of members of this clade have been well-characterized. Currently this clade is named for the *C. crescentus* ortholog that exhibited no phenotype when deleted (Zielińska et al., 2017).

*Agrobacterium tumefaciens* encodes one LmdC ortholog (Atu1832). The LmdC clade features an N-terminal transmembrane (TM) segment, one to two coiled coil domains, and an active LytM domain (Figure 1A). The LmdC clade has representatives from most classes but is particularly conserved in the Alpha- and Deltaproteobacteria and deep-branching proteobacteria (Figure 1B). This clade has few characterized representatives but is essential in *H. neptunium* where it could contribute to growth and division of daughter cells that bud from the reproductive prostheca (Cserti et al., 2017). It has also been implicated in prostheca (stalk) biogenesis in *C. crescentus* (Billini et al., 2019). Our phylogenetic analysis indicated that in all alphaproteobacterial orders, with the exception of Rhizobiales, the coiled-coil domain is preceded by a DUF5930 domain (Figure 1A). Overall, our analysis supports the idea that this gene is broadly conserved with specialized roles in the Alphaproteobacteria that has undergone further derivation in the Rhizobiales. To visualize the localization of this ortholog in *A. tumefaciens*, we constructed an cumate-inducible plasmid expressing LmdC*_*Atu*_* with monomeric superfolder GFP fused to its C-terminus. After inducing overnight, the cells showed low, diffuse fluorescence (Figure 2A). Attempts at fusing msfGFP at the native locus resulted in even lower levels of diffuse fluorescence (data not shown). An insertional knock-out of this gene exhibited no effect on cell viability or any morphological phenotype when grown in LB or ATGN (Figures 2E–G).

*Agrobacterium tumefaciens* encodes one MepM ortholog (Atu4178). This broadly conserved active LytM clade of DD-endopeptidases is generally distinguished by an N-terminal Csd3 inactivation domain (Figures 1A,B), which has been shown to obstruct the substrate binding site of the *Helicobacter pylori* MepM ortholog Csd3 in its crystal structure (An et al., 2015). MepM has been genetically associated with the elongasome in *E. coli* (Park et al., 2020) whereas the ortholog in the alphaproteobacterium *Sinorhizobium meliloti* (named “RgsM”) is a component of the polar elongasome (Krol et al., 2020). In agreement with *S. meliloti* and *A. tumefaciens* being close relatives and having polar elongation machinery in common, Atu4178 (RgsM*_*Atu*_*) had similar properties as RgsM*_*Sm*_*: RgsM*_*Atu*_*-msfGFP was mostly diffuse with some observable foci at mid-cell and at one pole (Figure 2B). After several attempts to delete RgsM*_*Atu*_*, we concluded it was essential, in agreement with its identification as an essential gene from high throughput transposon mutagenesis sequencing (Curtis and Brun, 2014). Thus, we constructed an RgsM depletion strain. In comparison to WT cells (Supplementary Video 1), depletion of RgsM inhibited polar elongation resulting in shortening of cells by 20 h (Figure 2E and Supplementary Video 2). Analysis of cell length indicated a significant decrease in cell length after depletion in comparison to WT or RgsM replete (+RgsM) cells (Figure 2F). Depletion of RgsM (−RgsM) results in a 5-log loss of viability confirming its essentiality (Figure 2G). The depletion of RgsM phenocopies the depletion of PBP1a, the major cell wall synthase driving polar elongation (Williams et al., 2021). These observations are consistent with characterization of RgsM*_*Sm*_* (Krol et al., 2020) suggesting that RgsM orthologs may function as a space-making DD-endopeptidases involved in the polar growth of Rhizobiales.

*Agrobacterium tumefaciens* conserves both divisome associated dLytM clades, EnvC (Atu2775) and NlpD (Atu1700). The EnvC protein architecture is characterized by a cleavable signal sequence, two or more coiled-coil domains, and an inactivated LytM domain (Figure 1A). The wide conservation of this gene suggests a common role in the proteobacterial divisome, where it activates cognate amidases, such as AmiAB in *E. coli* (Uehara et al., 2010) and AmiB in *V. cholerae* (Möll et al., 2014). EnvC*_*Atu*_*-msfGFP expressed from a plasmid under native promoter control localized to mid-cell during cell division, suggesting that this role is also conserved in *Agrobacterium* (Figure 2C). However, deleting *envC*_*Atu*_ resulted in shorter, hypercurved cells with a viability defect (Figures 2E–G and Supplementary Video 3). This phenotype differs from what has been reported for Δ*envC* strains in most other proteobacteria, where the deletion phenotype has been associated with cell separation defects, chaining, and often little to no viability loss (Hara et al., 2002; Uehara et al., 2009; Cserti et al., 2017; Zielińska et al., 2017).

Members of the NlpD clade contain a lipidation signal sequence for outer membrane anchoring, one or more PG-binding LysM domains that have been shown to help localize various NlpD orthologs (Goley et al., 2010; Möll et al., 2010; Poggio et al., 2010; Tsang et al., 2017), and a dLytM domain with a different inactivation pattern (two of the four chelating residues are generally conserved) (Figure 1A). In contrast to EnvC, this clade, as defined by its dLytM domain, appears to be constrained to the Alpha-, Beta-, and Gammaproteobacteria, while a few deep-branching proteobacteria have genes with a similar genetic architecture but active LytM domains (Figures 1A,B). NlpD has been extensively studied in *E. coli* where it activates AmiC and plays a role in outer membrane invagination during division (Uehara et al., 2009, 2010; Tsang et al., 2017). In *C. crescentus*, the NlpD ortholog “DipM” has been linked to cell separation and outer membrane invagination albeit with different morphological phenotypes than those typified by *E. coli* (Collier, 2010; Goley et al., 2010; Möll et al., 2010; Poggio et al., 2010; Zielińska et al., 2017). Expression of *dipM*_*Atu*_-msfGFP from a constitutive promoter on a pSRK plasmid indicated that localization was cell cycle dependent, localizing to mid-cell in pre-divisional and dividing cells and to the new pole in non-dividing cells (Figure 2D). This is a typical localization pattern for divisome factors in *A. tumefaciens*, such as FtsZ and FtsA (Howell et al., 2019), supporting DipM*_*Atu*_*’s role as a divisome component. However, we determined that *dipM* was essential, unlike NlpD orthologs in other proteobacteria. After multiple attempts to delete *dipM*, we constructed a depletion strain for DipM. Depletion of DipM resulted in filamented single cells with mid-cell bulges and loss of viability (Figures 2E–G and Supplementary Video 4). Similar to our observations for Δ*envC*, this phenotype was unexpectedly different from other proteobacteria, where NlpD is non-essential and the single mutant phenotype is either silent such as in *E. coli* (Uehara et al., 2009, 2010) and *V. cholerae* (Möll et al., 2014), or associated with cell separation defects such as in *N. gonorrhoeae* (Stohl et al., 2016), *H. influenzae* (Ercoli et al., 2015), or *C. crescentus* (Collier, 2010; Goley et al., 2010; Möll et al., 2010; Poggio et al., 2010; Zielińska et al., 2017). Given the uncharacteristic phenotypes of the divisome-associated LytM orthologs in *A. tumefaciens*, we focused on how these factors may function differently in this polar-growing Alphaproteobacterium.

### EnvC and AmiC Are Required for Maintenance of Rod Shape and Proper Polar Patterning

Instead of a cell separation defect, which is expected for an ortholog of an amidase activator that functions in the final steps of division, Δ*envC* cells exhibited hypercurvature that was readily detectable in both phase contrast (Figure 2E) and DIC (Figure 3A and Supplementary Video 3) in LB. Cells appeared curled and at times smaller than WT cells with no signs of cell separation defects. Similar, but less severe effects were seen in ATGN, a defined glucose-based medium (Supplementary Figures 4A,B). Although we were able to isolate Δ*envC* mutants on ATGN, the Δ*envC* strain exhibited very low viability in LB (Figures 2G, 3B) and suppressors arose fairly easily (data not shown), suggesting that EnvC plays a critical role in cell physiology. The morphological defects of Δ*envC* were complemented with a plasmid containing *envC* under native promoter control (Figures 3A,B). Although the cells did not exhibit observable cell separation defects, we tested other properties that have been associated with dLytM and amidase deletion strains in other proteobacteria, such as heightened susceptibility to detergents and salt concentrations (Möll et al., 2014; Zielińska et al., 2017). Δ*envC* was more sensitive to SDS and changes to salt concentrations (Figure 3C), suggesting that the defect may also include altered outer membrane stability and/or induce envelope stress. In addition, overproduction of EnvC resulted in a loss of viability (Figure 3D), suggesting it may activate cell wall hydrolases.

Given the data that EnvC localized to mid-cell during division and that Δ*envC* exhibited altered outer membrane stability, we used TEM to investigate the subcellular effects of the mutant. Whole cell TEM of Δ*envC* cells grown overnight indicated that the cells were hypercurved, bent, or kinked (Figure 3E). At low frequency in LB, some cells exhibited even more pleomorphic traits (Supplementary Figure 4C). The kinked or bent morphotypes were more frequently observed in Δ*envC* cells growing in ATGN as cells are generally longer and generate a more uniform rod morphology in this medium (Supplementary Figure 4D). In these cells, rather than being gradually curved along the entire cell length, the long axis appeared to be disrupted at one point that skewed the direction/alignment of elongation. This morphological defect may indicate that during the cell cycle, polar growth has become askew or misdirected from its normal polar trajectory that creates reliably straight rods. Thin section TEM indicated that cells did not exhibit any separation or outer membrane defects (Figure 3F), even in the most severe cases (see also Supplementary Figures 4E,F). Although dividing cells were rarely captured in our preparations of whole cells or thin sections, the few cases we did observe did not display any abnormalities in envelope structure (Supplementary Figure 4F). These data confirmed that loss of EnvC did not result in any typical cell separation defects, such as chaining or incomplete constriction of the PG and outer membrane layers, which is in marked contrast to what has been shown for other proteobacteria.

To observe how the Δ*envC* mutant grew and divided in real time, we performed time-lapse microscopy on cells growing on agar pads made with LB. The highly curved cell morphology made determination of division events difficult and hard to characterize (Supplementary Video 3). We were able to observe that the mutant displayed several defects in addition to hypercurvature that were generally associated with daughter cells (Figure 3G): a small percentage (about 10%) of cells elongated but did not appear to divide during the course of the experiment, indicating that division might be inhibited in a minority of cases. About 14% of the cells on the pad failed to elongate during the full course of the time-lapse experiment or after a division event, suggesting that despite division being possible, a portion of the population was unable to complete another cell cycle. Finally, about 25% of the cells exhibited a polarity defect: we were surprised to observe that after division, some daughter cells were seen to initiate elongation at the opposite pole than expected (Figure 3H). In WT cells, both the mother cell and daughter cell initiate growth at the pole produced by the division event, but in a subset of Δ*envC*_*Atu*_ cells, the daughter cells instead grew from the other pole (Figure 3G). Together, these data suggest that, unlike other proteobacteria, loss of EnvC in *A. tumefaciens* has clear morphological defects that are not due to defects in cell separation but may instead be due to disruption of early polar growth patterning.

Disruption of polar growth patterning has been linked to loss of the AmiC and EnvC orthologs in *S. meliloti* (Krol et al., 2021), leading us to wonder if this relationship was conserved in *A. tumefaciens*. Indeed, Δ*amiC* cells exhibited a similar hypercurved phenotype, decreased viability on LB, and increased sensitivity to SDS and salt (Figures 4A–C and Supplementary Video 5). The Δ*amiC* phenotype was complemented with a plasmid carrying *amiC* under T7-medium constitutive promoter control (Figures 4A,B). Time-lapse microscopy indicated that Δ*amiC* cells displayed the same distinct growth behavior on agarose pads as Δ*envC*_*Atu*_ (Figure 4D). The double mutant Δ*envC*Δ*amiC* exhibited a slightly more severe viability defect than Δ*amiC* but otherwise appeared to phenocopy the single mutants (Figures 3B, 4B,D and Supplementary Video 6). Together, this data suggested that, like in *S. meliloti*, EnvC and AmiC function in the same pathway.

We used CellTool (Pincus and Theriot, 2007) to run PCA on the mutant phenotypes to better compare them to WT and to each other (Supplementary Figure 5A). The largest deviation between the mutants and WT was attributed to cell curvature (shape mode 2, Figure 4E). Between mutants, the majority of the variance between Δ*envC* and Δ*amiC* was explained by cell length (shape mode 1, Supplementary Figure 5B). Both Δ*envC* and the double mutant were significantly shorter than WT and Δ*amiC*. Cell width (shape mode 3) contributed to a small amount of the morphological variance due to additive effects in the double mutant (Supplementary Figure 5C). While both single mutant strains were wider than WT, the double mutant strain was significantly wider than both. Overall, these differences support the idea that while EnvC and AmiC likely function in the same pathway (possibly by activation of AmiC by EnvC), EnvC appears to have additional roles in the cell and perhaps other regulatory partners.

All three mutants exhibited a similar polarity defect, in which the daughter cell that inherits the former growth pole initiates growth from either the new or old pole (Figures 4F,G). These observations are in agreement with data previously reported for *S. meliloti* (Krol et al., 2021). Like all alphaproteobacteria, *Agrobacterium* undergoes a dimorphic cell cycle with a non-motile mother cell that gives rise to a flagellated daughter cell. The cell cycle requires strict asymmetrical cellular patterning with unique poles. As a member of the polar-elongating Rhizobiales, during the cell cycle, the *Agrobacterium* mother cell grows polarly at the new pole, the machinery relocates to mid-cell to drive division, and the division event produces two new poles that are primed to be elongation poles (Figure 4G). In all three mutants, about half of the daughter cells do not elongate from the newly created pole after completing division, but rather from the opposite (old) pole.

Together, these strong converging phenotypes suggest that the EnvC ortholog may activate AmiC in *A. tumefaciens*, and, moreover, that *A. tumefaciens* can complete division without an amidase. AmpD (Atu2113) is the only other canonical amidase in the *A. tumefaciens* genome and has been previously shown to function in the PG recycling pathway (Figueroa-Cuilan et al., 2020). Moreover, Δ*ampD* had no morphological defects and the Δ*ampD*Δ*amiC* double mutant morphologically phenocopied Δ*amiC* (Supplementary Figures 6A–C). We hypothesize that EnvC may activate AmiC; however, we find that the absence of AmiC does not bypass the toxicity induced by EnvC overexpression (Supplementary Figure 6D), consistent with an additional role for EnvC beyond the putative regulation of AmiC. Remarkably, AmiC no longer appears to be necessary for cell separation in Rhizobiales genera and instead it predominantly functions to help establish growth pole patterning in the daughter cell.

### dLytM DipM Functions Early During Cell Division and Likely Intersects With the EnvC–AmiC Pathway

**FIGURE 5:**
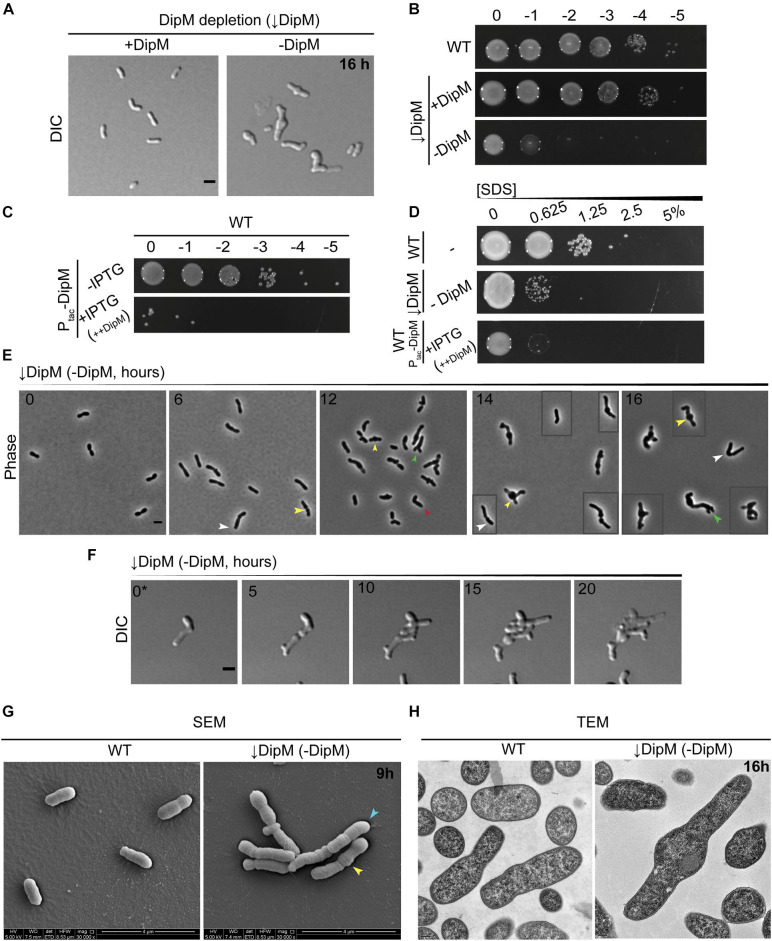
*dipM* is essential in *A. tumefaciens* and depletion results in mid-cell bulging, inhibition of division, and ectopic pole formation. **(A)** DIC imaging of DipM replete (↓DipM, +DipM; grown in the presence of IPTG) and depleted cells (↓DipM, –DipM) for 16 h. Depletion of DipM (↓DipM, –DipM) was achieved by washing the cells three times in LB and resuspending the culture (OD600 ∼ 0.1) in LB –IPTG. DipM depletion in LB results in mid-cell bulges and cell lysis. Scale bar = 2 μm. **(B)** Spot viability assays of WT, DipM replete (↓DipM, +DipM) and DipM depleted ((DipM, −DipM). Depletion of DipM (↓DipM, –DipM) was achieved by washing the cells three times in LB before spotting on LB plates. **(C)** Spot viability assays of WT cells containing a plasmid encoded DipM under the control of the Ptac promoter in the absence (–IPTG, uninduced) and presence [+IPTG, induced (++DipM)] of inducer driving expression from the plasmid. ++DipM indicates that DipM is being overproduced. **(D)** Outer membrane integrity was assessed during SDS sensitivity. WT cells are more resistant than the DipM depletion (↓DipM, –DipM) or DipM overproduction (+IPTG, ++DipM) strains. DipM was overexpressed (+IPTG, ++DipM) for 4 h before the SDS treatment and plated on LB plates. **(E)** Phase contrast images of DipM depleted cells (–DipM) for 0, 6, 12, 14, and 16 h in liquid LB. Arrowheads indicate main DipM depletion phenotypes: yellow arrowheads, mid-cell bulges; green arrowheads, tip-splitting events; white arrowheads, increased cell length; red arrowheads, bent/kinked cells. Boxed cells are additional examples of the field. Scale bar = 2 μm. **(F)** Time-lapse microscopy of DipM depleted cells on LB-agarose pads for 20 h. 0* = DipM cells were pre-depleted for 6 h before starting the time-lapse to avoid overcrowding. Boxed cells are spliced from elsewhere in the field to provide additional examples of diverse morphotypes. Time in hours is indicated on the image. Scale bar = 2 μm. **(G)** Scanning electron microscopy of WT and DipM depleted cells for 9 h (scale bar = 4 μm). Arrowheads indicate main DipM depletion phenotypes: yellow arrowheads, mid-cell bulges; blue arrowheads, cell filamentation. **(H)** Thin section transmission electron microscopy of WT and DipM-depleted cells for 16 h. Scale bar = 0.2 μm. All data collected for this figure was obtained from cells grown in LB.

Like all NlpD orthologs studied so far, DipM*_*Atu*_*-sfGFP localized to mid-cell during division (Figure 2A); unlike most NlpD orthologs studied so far, DipM*_*Atu*_* was essential. Depletion of DipM led to highly pleomorphic defects and lysis in most cells after 16 h of growth in LB and a strong cell viability defect (Figures 5A,B and Supplementary Video 4). DIC imaging of DipM-depleted cells showed that cells exhibited different combinations of mid-cell bulging, branching, overall loss of width control, and lysis by 16 h. The onset of these morphological effects is delayed in ATGN due to increased time for sufficient depletion of DipM; nevertheless DipM-depleted cells showed severe loss of viability on ATGN media in spot assays. Western blot analysis confirmed that the depletion of DipM was more rapid in LB than ATGN medium (Supplementary Figure 7A), presumably due to the faster growth rate.

To compare DipM to known properties of NlpD orthologs in other proteobacteria, we determined its overexpression phenotype and sensitivity to detergent. We overexpressed *dipM* by driving *dipM* expression from a plasmid under the pTAC promoter in WT cells. Overproduction of DipM severely reduced cell viability (Figure 5C), in agreement with the idea that it may activate hydrolytic factors such as shown in *Caulobacter* (Goley et al., 2010; Möll et al., 2010). While both depletion and overproduction of DipM resulted in loss of viability and lysis, these strains manifested lysis differently in that the overexpression strain did not exhibit the extreme pleomorphic traits of the depletion strain (Supplementary Figures 6C,E). Both the depletion strain and the overexpression strains were sensitive to SDS (Figure 5D), suggesting that both hypo- and hyperactivity of DipM regulation networks impact outer membrane stability or permeability. All of these observations are in line with what has been demonstrated in other proteobacterial genera (Goley et al., 2010; Möll et al., 2010, 2014; Tsang et al., 2017).

Because the depletion of DipM resulted in pleomorphic defects, we tracked the onset of changes in cell morphology and division over time using multiple approaches. Phase-contrast images of cells undergoing DipM depletion in liquid culture indicated morphological changes accumulate over time (Figure 5E): early on, cells elongated and often formed mid-cell bulges, and at later time points most cells appeared to be inhibited in division and had undergone both mid-cell bulging and various kinds of branching. Overall, these observations suggested that cells experienced loss of regulation of PG synthesis at mid-cell at low DipM levels and that in the absence of DipM, division was completely inhibited and the initiation of elongation was no longer strictly at the new pole. Next, we used time-lapse DIC microscopy to observe cells undergoing DipM depletion on agarose pads. In these experiments, we pre-depleted DipM for 6 h in liquid to avoid cell crowding caused by division events prior to sufficient DipM depletion (Figure 5F and Supplementary Video 4). It is likely that tracking the depletion in time-lapse allowed us to see many events and certain morphological trajectories that were more vulnerable to lysis and lost in sample handling for liquid culture time points. We found that each cell had its own unique trajectory that made it hard to generalize this phenotype and have included one example in Figure 5F to outline some major themes (see also Supplementary Video 4, for other examples). Of note, the cell in Figure 5F never terminated polar growth before division events, leading to tip-splitting and bipolar growth (see daughter cell behavior at 10, 15 h), formed ectopic poles at mid-cell bulges (see mother cell behavior at 10, 15h), and underwent erratic and atypical division events. The different types of branching (tip splitting or ectopic pole formation) observed during DipM depletion have been observed previously: tip splitting with FtsZ depletions and branching from mid-cell with FtsA and FtsW depletions (Howell et al., 2019). Moreover, bulging at mid-cell occurs in the absence of FtsW or both PBP3a and PBP3b and has been interpreted as an indicator of inhibition or misregulation of sPG synthesis (Howell et al., 2019; Williams et al., 2021). The combination of these defects in the depletion of DipM suggests a central role for DipM in the redirection of PG synthesis to mid-cell and subsequent cell division.

We used various forms of electron microscopy to determine the effect of the DipM depletion at the subcellular level. TEM and SEM of whole cells indicated a similar pattern to that seen in phase-contrast and DIC images of cells undergoing DipM depletion (Figure 5G and see also Supplementary Figures 7B,C). At 8 h, we observed particularly long cells that exhibited multiple bulges, suggesting that if these bulges are mid-cell bulges, these cells might have scaffolded more than one division plane (mid-cell region) over the course of their cell cycles but failed to divide (Supplementary Figure 7B). SEM of cells depleted for 9 h indicated rounds of failed constriction as well as multiple mid-cell bulges (Figure 5G and Supplementary Figure 7D). The accumulation of phenomena associated with the division plane suggests that DipM may play an indirect role in early division, such as stabilizing the divisome, and that depleting DipM may indirectly destabilize nascent, constricting Z-rings.

It seemed possible that some of the branched cellular filaments created by depleting DipM could consist of chained cells, or that areas with constrictions might have completed cytokinesis of the inner membrane but were still linked by PG and the outer membrane. However, thin section TEM of cells at various time points indicated that even cells with massive morphological defects did not exhibit any abnormalities in their envelope layers along the cell length or at division sites (Figure 5H and see also Supplementary Figure 7C). Slices containing cells sectioned parallel to their long axis did not reveal any internal compartments separated by IM layers, verifying that cell chaining did not underlie the filamenting phenotype. These data indicate that the predominant effect of DipM depletion in *A. tumefaciens* was inhibition of division during early steps of constriction and not the final steps of septation, as shown for other proteobacteria.

**FIGURE 6:**
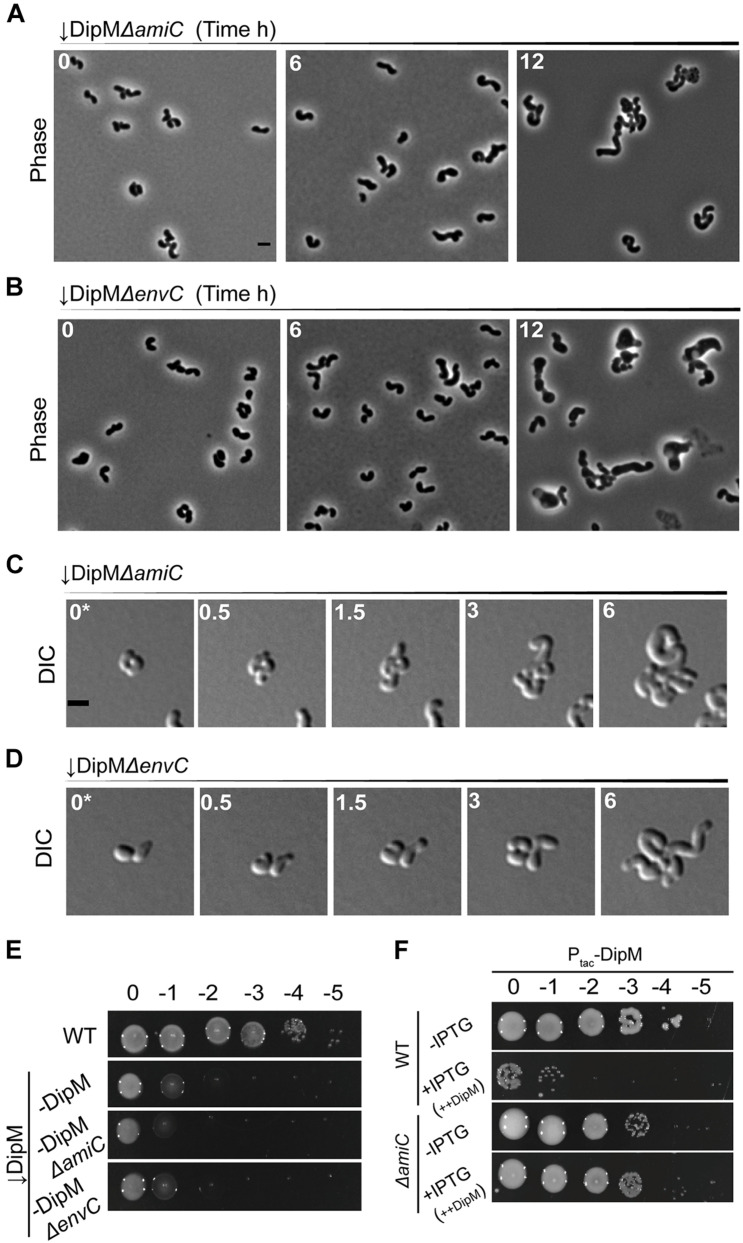
DipM likely functions in pathways that are distinct from, but overlap with, the EnvC–AmiC pathway. Phase-contrast microscopy of **(A)** ↓DipMΔ*amiC* or **(B)** ↓DipMΔ*envC* depleted for exactly 0, 6, and 12 h. **(C)** DIC time-lapse of ↓DipMΔ*amiC* or **(D)** ↓DipMΔ*envC* pre-depleted for 6 h in liquid to avoid overcrowding caused by division events prior to sufficient DipM depletion. **(E)** Spot viability assays of WT, DipM depleted (–DipM), –DipMΔ*amiC*, and –DipMΔ*envC* cells. **(F)** Cell viability of WT and Δ*amiC* cells containing empty plasmid (Ptac-Empty) or overproducing DipM (+IPTG, ++DipM). ++DipM indicates that DipM is being overproduced. To better define the *dipM* overexpression phenotype in WT and Δ*amiC*, spotting assays were conducted in ATGN (minimal medium). All data collected for this figure was obtained from cells grown in LB.

Because the defects from depleting DipM could be associated with misregulation of septal hydrolases and because AmiC is directly regulated by the NlpD ortholog in *E. coli* (Uehara et al., 2010), we sought to determine any possible connection between these two genes in *A. tumefaciens*. We first wanted to determine if loss of AmiC would alleviate any of the manifold morphological defects of the DipM depletion. Depleting DipM in the Δ*amiC* background resulted in bloated, curved cells that appeared to exhibit a mixture of the two phenotypes with no improved outcomes in filamentation or viability (Figures 6A,C,E and Supplementary Video 7). In both time points of liquid culture and in time-lapse observations, it seemed possible that the mid-cell bulges were non-existent or reduced; however, the cumulative effects on cell width made this possible effect hard to determine. We also depleted DipM in the Δ*envC* background to determine if there were any EnvC-specific effects on the DipM depletion phenotype. The outcome was similar to, if not worse than, depleting DipM in the Δ*amiC* background, with additive effects on loss of width control (Figures 6B,D,E and Supplementary Video 8). In both cases, the double mutants appeared to exhibit a combination of the predominant features of Δ*amiC*/Δ*envC* and the DipM depletion.

The absence of EnvC or AmiC pathways exacerbated the DipM depletion phenotype suggesting that DipM functions in a pathway distinct from EnvC–AmiC or other EnvC pathways. We were therefore surprised to find that loss of AmiC completely alleviated the toxicity of *dipM* overexpression (Figure 6F and Supplementary Figure 6D). The absence of AmpD was not sufficient to relieve the toxicity of *dipM* overexpression (Supplementary Figures 6C,D). These results suggest that the toxic effects of *dipM* overexpression stem predominantly from crosstalk with the EnvC–AmiC pathway and is unrelated to its roles in regulating division or termination of elongation. Therefore, there is some overlap between the DipM and EnvC–AmiC pathways, but DipM’s other roles in division and regulation of termination of elongation obscure this relationship during DipM depletion.

## Discussion

**FIGURE 7:**
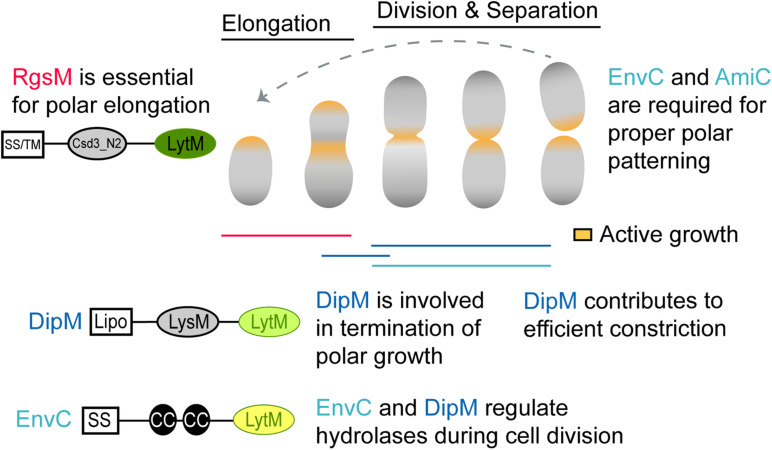
Schematic of proposed LytM factor functions in *A. tumefaciens*. During polar elongation, the essential RgsM likely functions at the new pole, where it likely modifies the cell wall to allow the addition of new PG material. DipM also localizes to the new pole during polar elongation and polar elongation often fails to terminate in absence of DipM, suggesting that DipM may interact with the elongasome and cell wall hydrolases to help regulate unipolar growth. In the absence of DipM the redirection of growth to mid-cell occurs but frequently leads to mid-cell bulges rather than cell division suggesting that DipM likely regulates PG hydrolases involved in early stages of cell division. Finally, EnvC regulates the activity of AmiC at mid-cell during cell division. While this is a canonical function for a dLytM factor, EnvC and AmiC are not required for cell separation but rather for proper establishment of growth poles following cell division. Protein schematics are shown as described in Figure 1.

LytM domain-containing proteins are widespread in the bacterial domain and have evolved to occupy many roles, most of which are still unknown. Of particular interest in this enzyme family is the frequency at which the catalytic site of this domain has been reconfigured and co-opted for non-enzymatic, regulatory purposes. Using bioinformatics, we determined that *A. tumefaciens* putatively encodes two enzymatically active LytM factors and two inactive (dLytM) factors. By constructing a gene tree of LytM factors from the Proteobacteria and their deep-branching relatives, we showed that these four LytM factors fall into four of five major clades that have orthologs and paralogs in other proteobacterial genera. Although the LmdC ortholog exhibited no clear localization pattern or null phenotype, the MepM ortholog exhibited weak polar and mid-cell localization and was an essential polar elongation factor (Figures 2, 7). This finding is in agreement with what has been shown for the MepM ortholog “RgsM” in *S. meliloti* (Krol et al., 2020), a close, polar-growing relative of *A. tumefaciens*. Both dLytM factors, EnvC*_*Atu*_* and DipM*_*Atu*_*, localized to mid-cell during cell division, in agreement with most studies in proteobacteria that have shown that these factors are divisome components. However, neither dLytM depletion or deletion gave rise to expected cell separation defects in *Agrobacterium* despite consensus of cell separation defects reported in the literature for alphaproteobacterial genera (Cserti et al., 2017; Meier et al., 2017; Zielińska et al., 2017). Cell separation defects due to interrupting the Tol–Pal system have been observed in Rhizobiales member *S. meliloti*, where depletion of TolQ resulted in OM blebbing and envelope defects at the division plane (Krol et al., 2020). Neither Δ*envC* nor the DipM depletion in *A. tumefaciens* manifested as cell separation phenotypes.

Interpretation of phenotypes resulting from deletion/depletion of dLytMs in alphaproteobacterial members is challenging because divisome components potentially impact distinctly different cell cycles and growth modes in these genera. Given that *A. tumefaciens* is a polar-growing alphaproteobacterium, we expect division mutants to have distinct morphological phenotypes from other proteobacteria even if they maintain canonical function. For example, *A. tumefaciens* conserves many essential divisome components such as FtsZ, FtsA, and FtsW. Instead of forming long, smooth filaments typical of laterally elongating proteobacteria, the depletion of these factors results in a division defect unique to polar-growing bacteria. Depletion of FtsZ results in branched cells that accumulate growth poles, suggesting that FtsZ is required for redirection of growth machinery to mid-cell (Howell et al., 2019). In contrast, absence of FtsA or FtsW leads to continuous redirection of growth to mid-cell during each subsequent cell cycle. Since division has not occurred, ectopic growth poles form resulting in an asterisk-shaped cell that ultimately lyses (Howell et al., 2019). Given this framework, it is important to carefully interpret the dLytM mutants we observed in this study. We must determine whether the atypical phenotypes we observed are due to (1) the polar elongation context alone, (2) derived roles for these factors in polar growth in the Rhizobiales, or even (3) ancient and shared roles of these factors that are more evident in this genetic background than other proteobacteria. Although this task will ultimately require many more studies, there are some hypotheses we can make with our current observations.

Because the DipM depletion resulted in a clear division defect, we will consider it first. DipM was unexpectedly essential, and inspection of DipM-depleted cells explained why: depletion of DipM resulted in cells with inefficient termination of polar growth and critical deficiencies in the regulation of sPG synthesis resulting in multiple rounds of attempted cell division. These observations suggest that DipM plays multiple roles in *A. tumefaciens* (Figure 7). Some roles may be the same as already demonstrated in other proteobacteria, where NlpD orthologs have been shown or suggested to regulate the activity of hydrolases besides amidases (Uehara et al., 2009; Möll et al., 2014; Zielińska et al., 2017). Misregulation of hydrolases and/or autolysins at mid-cell could explain the mid-cell bulging phenotype. We can also attribute some roles to derived functions for Rhizobiales, as the loss of termination of elongation during the cell cycle was pronounced in many of the cells we observed in time-lapse. Because the types of branching (tip-splitting vs. formation of ectopic poles at mid-cell) were varied, it is possible that this is an indirect effect *via* interactions with the divisome or elongasome. Indeed, this brings us to the final point, that the clear marks of initiated but failed rounds of constriction in the DipM-depleted cells suggests that DipM may indirectly help stabilize the nascently constricting divisome. This final role may be shared by other proteobacteria as Δ*dipM* was shown to perturb FtsZ behavior in *Caulobacter* (Poggio et al., 2010) and *Pseudomonas* Δ*nlpD* cells exhibited early inhibition of constriction in cell filaments (Yakhnina et al., 2015). We conclude that the NlpD ortholog of *A. tumefaciens* functions in conserved, derived, and potentially undetected ancestral roles.

While *envC* was not essential in *A. tumefaciens*, its loss greatly reduced cell viability. Δ*envC* cells were hypercurved and somewhat smaller than WT cells. Time-lapse microscopy revealed that a subset of the Δ*envC* population aberrantly initiated elongation at the old pole. Quantification of these defects suggested that they occur primarily in the daughter cells, perhaps suggesting that the loss of viability in this mutant is due to the production of a significant number of non-viable daughter cells. Therefore, the primary function of EnvC in *A. tumefaciens* appears to lie in its role in polar growth patterning (Figure 7). We found that AmiC also appears to function in this pathway, as its deletion strain and double mutant with EnvC generally phenocopy Δ*envC*. Krol et al. (2021) have demonstrated similar phenomena in *S. meliloti*, and have further shown that the EnvC–AmiC pathway facilitates the accumulation of the FtsN-like protein RgsS at mid-cell, presumably by binding amidase-processed PG at the septum. Overall, these observations suggest a derived function for EnvC in the Rhizobiales in which the AmiC pathway has at least been partially co-opted for polar growth patterning. Like DipM, however, EnvC appeared to function in multiple pathways, suggesting that while it has been co-opted, it may also still maintain some ancestral functions that are masked by the dominant polarity defects. The connection between EnvC and AmiC has been suggested in genetic studies of other Alphaproteobacteria. However, in *C. crescentus*, *in vitro* assays showed that the dLytM domain of NlpD*_*Cc*_* (“DipM”) mildly stimulated AmiC*_*Cc*_* rather than that of EnvC*_*Cc*_* (“LdpF”) (Meier et al., 2017), as expected from similar experiments for *E. coli* orthologs. Therefore the molecular details of this putative EnvC–AmiC pathway might not resemble the EnvC–AmiAB regulatory mechanism demonstrated in *E. coli* (Uehara et al., 2010) and needs further study in *A. tumefaciens*.

Our phylogenetic approach allowed us a large, if coarse-grained, view of LytM domain evolution. By collecting LytM domain-containing proteins from various proteobacterial and deep-branching proteobacterial relatives, we were able to distinguish five different LytM clades. The short length of the LytM domain (110–120 amino acids) does not give enough signal for confidence in the branching order and therefore the evolutionary relationships of these clades (Supplementary Figure 2). Within this study, we can only reliably infer which orthologs a bacterium encodes. It is also important to note that our current study only captured a subset of LytM families and it is likely that with enough sampling of deep-branching proteobacteria, many of the unclassified LytM proteins (thin branches in Figure 1A and classified as “other” in Figure 1B) would form distinct clades. It is clear that the LytM domain, in both its enzymatic and inactivated forms, has evolved to participate in many different pathways in different classes or genera. Although our analysis has categorized these genes into five major clades, this classification in no way suggests a shared functional role, only a shared ancestral past. For example, the Csd LytM factors that play a role in the helical morphology of *H. pylori* have arisen from both the MepM (Csd3) and LmdC (Csd1 and Csd2) clades (An et al., 2015, 2016; Yang et al., 2019). The MepM ortholog of *N. gonorrhoeae*, Mpg, has been shown to be a virulence factor required for pilus biogenesis and natural competence (Stohl et al., 2012, 2013).

Even clades that we suppose are more constrained by critical roles in division, namely the dLytM clades, have members that have been shown to exhibit derived (class or species-specific) functions, such as influencing protein secretion in *H. influenzae* (Ercoli et al., 2015), T3S apparatus assembly in *X. campestris* (Yang et al., 2018), and stalk biogenesis in *C. crescentus* (Billini et al., 2019). While we cannot determine a branching order for the EnvC and NlpD classes with great confidence using our current approach, using the LytM domain tree in combination with the conserved N-terminal domains makes it possible to infer that EnvC and NlpD were likely inactivated independently from each other. The inactivated enzymatic site itself has been shown to participate in autoinhibitory regulation of both EnvC and AmiB (Peters et al., 2013; Cook et al., 2020), making it an interesting motif to track in both NlpD and EnvC lineages. In general, NlpD orthologs conserve two of the signature residues and have at least one conserved substitution [**N**XXXD (**K**/X)XH] whereas EnvC orthologs conserve none of the signature residues and also exhibit distinct substitution patterns among bacterial classes [(**W**/X)XXX, XX**Y]** (Supplementary Figure 2). The conserved yet distinct substitutions in both of these clades reflects their divergence, potentially driven by coevolution with amidases or other potential regulatory partners. In support of this argument, the tryptophan (W) and tyrosine (Y) substitutions in the catalytic site of EnvC have been shown to be required for the activation of cognate amidase AmiB in *E. coli* (Peters et al., 2013). Conservation of these clade-specific substitutions suggests overall maintenance of regulatory partners among various proteobacteria.

Nevertheless, the data from different proteobacterial species seem to suggest that the relationship between dLytMs and their cognate amidases could be plastic: in some cases, a dLytM has been shown to activate more than one amidase, such as ActS in *E. coli* (Gurnani Serrano et al., 2021); in others multiple dLytM factors are thought to activate one amidase, such as in *V. cholerae* (Möll et al., 2014) or *P. aeruginosa* (Yakhnina et al., 2015); and in still others, dLytM factors could activate a different amidase than in *E. coli*, such as could be the case in *H. neptunium* (Cserti et al., 2017), *S. meliloti* (Krol et al., 2021), and *A. tumefaciens*. Our limited analysis of 178 LytM domains does not indicate for any of these aforementioned species that their dLytM domain sequences contain substitutions that would suggest shifts in protein partners or function. For example, if Rhizobiales dLytM genes had evolved entirely different roles from other proteobacteria, we expect them to form distinct clusters away from the other alphaproteobacteria on the tree, such as how the EnvC orthologs in the Epsilonproteobacteria form a cluster distinct from the rest of all of the EnvC orthologs, or the Rhodobacterales NlpD orthologs form a distinct cluster from the other alphaproteobacterial NlpD orthologs (Figure 1A). While the dLytM domains themselves do not indicate strong evidence of positive selection for new partners or function, it is much more likely that the N-terminal domains in the full length LytM genes may be the areas where diversification and subfunctionalization would take place. In fact, in *V. cholerae*, where both dLytM factors appear to regulate AmiB*_*Vc*_*, the LytM domain of NlpD*_*Vc*_* does not appear to be necessary for this activation (Möll et al., 2014). This may indicate that amidases could be activated by LytM domains in ways that are much more diverse than currently appreciated.

In summary, we have taken the first steps to characterize the LytM factors of *A. tumefaciens*. The dLytM factors were particularly interesting in that their loss gave rise to unexpected phenotypes that suggest they both operate as regulatory hubs with potentially many partners to influence distinct and overlapping processes during the cell cycle (Figure 7). Although the essentiality of DipM made it difficult to assess the potential intersection of these pathways, our preliminary observations that the mid-cell bulging during DipM depletion and the toxicity of *dipM* overexpression were alleviated in the Δ*amiC* strain support this idea. The implication of both of these factors in polar processes might suggest that their dominant functions are derived for polar growth regulation in Rhizobiales, however, that might not necessarily be true. Deletion of DipM in *C. crescentus* resulted in mislocalization of polarity factor PopZ to both poles (Möll et al., 2010). Deletion of FtsE, the recruiter of EnvC, in *C. crescentus* resulted in thin cellular connections between mother and daughter cells that accumulated stalk biogenesis factors and stalk features (Meier et al., 2017). Together, these could suggest that dLytM factors play roles in influencing polarity in alphaproteobacteria in general. Finally, although polarity is not generally studied in bacteria that divide symmetrically, such as the Gamma- and Betaproteobacteria, studies have indicated that molecular polarity in *E. coli* manifests in aging effects (Lindner et al., 2008) and polar cytoplasmic features, such as chemosensory arrays (Rajendran et al., 2014; Santos et al., 2014; Oh et al., 2018). It is our hope that our work in a more recognizably polar bacterium inspires others to look for polarity and factors that may influence it in their own systems.

## Data Availability Statement

The original contributions presented in the study are included in the article/Supplementary Material, further inquiries can be directed to the corresponding author.

## Author Contributions

WF-C and AR were responsible for conceptualization, formal analysis, investigation, methodology, validation, visualization, writing the original draft, and reviewing and editing. CD, GS-C, and AY contributed to the investigation. AR and PB acquired funding to support this research. PB contributed to conceptualization, project administration, resources, supervision, and editing and revising. All authors contributed to the article and approved the submitted version.

## Conflict of Interest

The authors declare that the research was conducted in the absence of any commercial or financial relationships that could be construed as a potential conflict of interest.

## Publisher’s Note

All claims expressed in this article are solely those of the authors and do not necessarily represent those of their affiliated organizations, or those of the publisher, the editors and the reviewers. Any product that may be evaluated in this article, or claim that may be made by its manufacturer, is not guaranteed or endorsed by the publisher.
