# Supplementary material for: Diversification of LytM Protein Functions in Polar Elongation and Cell Division of Agrobacterium tumefaciens
Source: Front Microbiol. 2021 Aug 18;12:729307. doi: 10.3389/fmicb.2021.729307 (PMC8416486; doi:10.3389/fmicb.2021.729307)
Supplement: Supplementary file 1 [file Data_Sheet_1.pdf]

Supplementary Material

Diversification of LytM protein functions in polar elongation and cell division of *Agrobacterium tumefaciens*

Wanda M. Figueroa-Cuilan [1+^], Amelia M. Randich[2+], Caroline Dunn[1], Gustavo Santiago-Collazo[1,3], Andrew Yowell[1], and Pamela J.B. Brown[1*]

[1]Division of Biological Sciences, University of Missouri-Columbia, Columbia, MO, USA

[2]Department of Biology, University of Scranton, Scranton, PA, USA

[3]Molecular Therapeutics and Pathogenesis Graduate Program, University of Missouri-Columbia, Columbia, MO, USA

[+]denotes equal contributions

Current Addresses:

[^]Department of Biological Chemistry, Johns Hopkins University School of Medicine, Baltimore, MD, USA

[$]Department of Biology, Indiana University, Bloomington, IN, USA

[&]University of Iowa, College of Dentistry and Dental Clinics, Iowa City, IA, USA

*** Correspondence:**

Pamela Brown

brownpb@missouri.edu

1. Supplementary Tables

Supplementary Table 1. LytM gene, genome accession numbers, and data.

IMG Gene ID	Clade	Catalytic Site	SS/LIPO /TM [a]	Other domains [b]	Locus Tag	IMG Genome ID	Genome Name	Class
637055808	Other	RDHH	SS	DUF4121	PA5363	637000218	Pseudomonas aeruginosa PAO1	Gammaproteobacteria
637088345	Other	HDHH	LIPO		CC2297	637000061	Caulobacter vibrioides CB15	Alphaproteobacteria
637089355	Other	HDHH	no TM		CC3301	637000061	Caulobacter vibrioides CB15	Alphaproteobacteria
638022820	Other	HDHH	LIPO		MXAN_1433	637000186	Myxococcus xanthus DK 1622	Deltaproteobacteria
638023451	Other	HDHH	no TM		MXAN_2075	637000186	Myxococcus xanthus DK 1622	Deltaproteobacteria
638024911	Other	HDHH	TM		MXAN_3554	637000186	Myxococcus xanthus DK 1622	Deltaproteobacteria
638028210	Other	-DHH	no TM		MXAN_6905	637000186	Myxococcus xanthus DK 1622	Deltaproteobacteria

638145539	Other	HDHH	LIPO		HNE_2982	637000135	Hyphomonas neptunium ATCC 15444	Alphaproteobacteria
638145966	Other	HDHH	LIPO		HNE_3409	637000135	Hyphomonas neptunium ATCC 15444	Alphaproteobacteria
648173582	Other	HDHH	SS	LysM	STHERM_c07180	648028053	Spirochaeta thermophila DSM 6192	Spirochaetia
648173610	Other	HDHH	TM	2xLysM	STHERM_c07460	648028053	Spirochaeta thermophila DSM 6192	Spirochaetia
648175072	Other	EDHH	SS		STHERM_c22090	648028053	Spirochaeta thermophila DSM 6192	Spirochaetia
649960984	Other	HDHH	TM		Odosp_1646	649633078	Odoribacter splanchnicus 1651/6, DSM 20712	Bacteroidia
2540578544	Other	HDHH	TM	CC	LFE_1702	2540341086	Leptospirillum ferrooxidans C2-3	Nitrospira
2563183931	Other	ADHH	LIPO	no CC	XALC_2187	2562617136	Xanthomonas albilineans GPE PC73	Gammaproteobacteria

2578483098	Other	HDHH	SS	no CC	EP01_11365	2576861479	Bdellovibrio bacteriovorus 109J	Deltaproteobacteria
2804931142	Other	HDMH	SS		Ga0263390_11406	2802429312	Halobacteriovorax marinus BE01	Deltaproteobacteria
2832892736	Other	RDHH	SS		Ga0335562_174	2832892563	Salinimonas sediminis N102	Gammaproteobacteria
2848965275	Other	HNQQ	TM		Ga0349804_01_ 1040985_1041854	2848964341	Flavobacterium album HYN0059	Flavobacteriia
637051048	MepM	HDHH	no TM	OapA, Csd3_N2	PA0667	637000218	Pseudomonas aeruginosa PAO1	Gammaproteobacteria
637089086	MepM	HDHH	TM	Csd3_N2	CC3034	637000061	Caulobacter vibrioides CB15	Alphaproteobacteria
637142884	MepM	HDHH	SS (low)/TM		NGO1686	637000188	Neisseria gonorrhoeae FA 1090	Betaproteobacteria
637183429	MepM	HDHH	TM		SMc02432	637000269	Sinorhizobium meliloti 1021	Alphaproteobacteria
637477582	MepM	HDHH	TM	Csd3_N2	RPA4431	637000239	Rhodopseudomon as palustris CGA009	Alphaproteobacteria
638143205	MepM	HDHH	TM		HNE_0632	637000135	Hyphomonas neptunium ATCC 15444	Alphaproteobacteria

638143206	MepM	HDHH	TAT (low)/TM	Csd3_N2	HNE_0633	637000135	Hyphomonas neptunium ATCC 15444	Alphaproteobacteria
639299496	MepM	HDHH	TM	Csd3_N2	Atu4178	639279302	Agrobacterium tumefaciens C58-UWash	Alphaproteobacteria
640673661	MepM	HDHH	no TM	Csd3_N2	CGSHi22121_07155	640612215	Haemophilus influenzae NTHi 22.1-21, ST176	Gammaproteobacteria
640819612	MepM	HDHH	SS		NIS_0343	640753037	Nitratiruptor sp. SB155-2	Epsilonproteobacteria
643382839	MepM	HDHH	TM	csd3_N, Csd3_N2	HPG27_464	643348558	Helicobacter pylori G27	Epsilonproteobacteria
643724394	MepM	HDHH	SS	Csd3_N, Csd3_N2	NAMH_0648	643692029	Nautilia profundicola Am-H	Epsilonproteobacteria
644795258	MepM	HDHH	no TM	Csd3_N2	Vapar_4673	644736413	Variovorax paradoxus S110	Betaproteobacteria
648147843	MepM	HDHH	TM	Csd3_N2	Galf_0360	648028028	Gallionella capsiferriformans ES-2	Betaproteobacteria
649962289	MepM	HDHD	TM	Csd3_N2	Odosp_2968	649633078	Odoribacter splanchnicus 1651/6, DSM 20712	Bacteroidia

2508710873	MepM	HDHH	no TM (weak)	Csd3_N2	Dsui_1335	2508501046	Azospira oryzae PS	Betaproteobacteria
2511844843	MepM	HDHY	SS		F11_02585	2511231162	Rhodospirillum rubrum F11	Alphaproteobacteria
2511845117	MepM	HDHH	SS (low)	Csd3_N2	F11_03950	2511231162	Rhodospirillum rubrum F11	Alphaproteobacteria
2563182133	MepM	HDHH	TM	OapA, Csd3_N2	XALC_0382	2562617136	Xanthomonas albilineans GPE PC73	Gammaproteobacteria
2600368686	MepM	HDHH	TM	OapA_N, LysM, Csd3_N2	Ga0063390_00146	2600254932	Escherichia coli K-12 MG1655	Gammaproteobacteria
2630389325	MepM	HDHH	TM	Csd3_N2	Ga0078022_11808	2627854142	Nitrosomonas communis Nm2	Betaproteobacteria
2645939808	MepM	HDHH	LIPO	OapA, Csd3_N2	Ga0100913_1168	2645727549	Vibrio cholerae O1	Gammaproteobacteria
2645942972	MepM	HDHH	TM	OapA_N, Csd3_N2	Ga0100913_122285	2645727549	Vibrio cholerae O1	Gammaproteobacteria
2646276661	MepM	HDHD	TM	Csd3_N2	Ga0046402_113192	2645727629	Desulfovibrio vulgaris RCH1	Deltaproteobacteria
2649937682	MepM	HDHH	SS	Csd3_N	Ga0077940_11324	2648501445	Sulfurovum lithotrophicum ATCC BAA-797	Epsilonproteobacteria

2668358420	MepM	HDHH	SS	Csd3_N, Csd3_N2	Ga0110964_111228	2667527488	Campylobacter jejuni CJ677CC008	Epsilonproteobacteria
2689559549	MepM	HDHH	SS	Csd3_N, Csd3_N2	Ga0133481_11408	2687453639	Helicobacter typhlonius 1	Epsilonproteobacteria
2721281612	MepM	HDHH	SS	Csd3_N2	Ga0174992_112500	2718218393	Chromobacterium vaccinii 21-1	Betaproteobacteria
2774609376	MepM	HDHH	TM	Csd3_N2	Ga0226477_11584	2773857813	Pasteurella dagmatis NCTC 11617	Gammaproteobacteria
2776475379	MepM	HDHH	TM	Csd3_N2	Ga0263277_111259	2775506949	Mariprofundus ferrinatatus CP-8	Zetaproteobacteria
2804932538	MepM	HDHH	LIPO	Csd3_N2	Ga0263390_111803	2802429312	Halobacteriovorax marinus BE01	Deltaproteobacteria
2812896561	MepM	HDHH	no TM	Csd3_N2	Ga0213641_113265	2811995033	Comamonas aquatica CJG	Betaproteobacteria
2832895623	MepM	HDHH	SS	OapA, Csd3_N2	Ga0335562_3061	2832892563	Salinimonas sediminis N102	Gammaproteobacteria
2832957786	MepM	HDHH	no TM	OapA, Csd3_N2	Ga0335639_1724	2832956063	Salinisphaera sp. LB1	Gammaproteobacteria
2832958155	MepM	HDHH	SS	Csd3_N2	Ga0335639_2093	2832956063	Salinisphaera sp. LB1	Gammaproteobacteria

2832959154	MepM	HDHL	SS	Csd3_N2	Ga0335639_3092	2832956063	Salinisphaera sp. LB1	Gammaproteobacteria
2848965119	MepM	HDHC	LIPO	Csd3_N2	Ga0349804_01_865078_866316	2848964341	Flavobacterium album HYN0059	Flavobacteriia
638027156	MepM	HDHH	SS		MXAN_5829	637000186	Myxococcus xanthus DK 1622	Deltaproteobacteria
637055576	EnvC	WLAY	SS		PA5133	637000218	Pseudomonas aeruginosa PAO1	Gammaproteobacteria
637089488	EnvC	STEY	SS		CC3434	637000061	Caulobacter vibrioides CB15	Alphaproteobacteria
637141847	EnvC	WFGY	SS		NGO0571	637000188	Neisseria gonorrhoeae FA 1090	Betaproteobacteria
637183998	EnvC	LMTY	SS	2XCC	SMc03782	637000269	Sinorhizobium meliloti 1021	Alphaproteobacteria
637473293	EnvC	ESVY	SS	ATG16	RPA0168	637000239	Rhodopseudomonas palustris CGA009	Alphaproteobacteria
638027073	EnvC	QDYY	SS		MXAN_5746	637000186	Myxococcus xanthus DK 1622	Deltaproteobacteria
638145767	EnvC	ASET	SS		HNE_3210	637000135	Hyphomonas neptunium ATCC 15444	Alphaproteobacteria

639298261	EnvC	SVTY	SS		Atu2775	639279302	Agrobacterium tumefaciens C58-UWash	Alphaproteobacteria
640673221	EnvC	WVAY	LIPO	2X CC	CGSHi22121_04940	640612215	Haemophilus influenzae NTHi 22.1-21, ST176	Gammaproteobacteria
640820041	EnvC	NEKI	LIPO	2XCC	NIS_0757	640753037	Nitratiruptor sp. SB155-2	Epsilonproteobacteria
643383075	EnvC	STRG	no TM	2X CC	HPG27_707	643348558	Helicobacter pylori G27	Epsilonproteobacteria
643724266	EnvC	NTAE	SS (low)	2XCC	NAMH_0520	643692029	Nautilia profundicola Am-H	Epsilonproteobacteria
648147693	EnvC	WFGY	SS		Galf_0211	648028028	Gallionella capsiferriformans ES-2	Betaproteobacteria
2508710809	EnvC	WFGY	SS (low)	ATG16	Dsui_1271	2508501046	Azospira oryzae PS	Betaproteobacteria
2511845610	EnvC	LRTY	TAT		F11_06380	2511231162	Rhodospirillum rubrum F11	Alphaproteobacteria
2563182143	EnvC	SLAY	LIPO (low)		XALC_0392	2562617136	Xanthomonas albilineans GPE PC73	Gammaproteobacteria

2578483389	EnvC	HFGY	SS		EP01_12830	2576861479	Bdellovibrio bacteriovorus 109J	Deltaproteobacteria
2600370481	EnvC	WVSY	SS		Ga0063390_01945	2600254932	Escherichia coli K-12 MG1655	Gammaproteobacteria
2630391201	EnvC	WFGY	SS (low)		Ga0078022_112687	2627854142	Nitrosomonas communis Nm2	Betaproteobacteria
2645943266	EnvC	WVAY	SS		Ga0100913_122581	2645727549	Vibrio cholerae O1	Gammaproteobacteria
2646275660	EnvC	VGGY	SS		Ga0046402_112186	2645727629	Desulfovibrio vulgaris RCH1	Deltaproteobacteria
2649938043	EnvC	NTKM	LIPO	2XCC	Ga0077940_11685	2648501445	Sulfurovum lithotrophicum ATCC BAA-797	Epsilonproteobacteria
2668358482	EnvC	NVDT	SS	DUF3450	Ga0110964_111290	2667527488	Campylobacter jejuni CJ677CC008	Epsilonproteobacteria
2689560779	EnvC	NIRS	SS	2XCC	Ga0133481_111639	2687453639	Helicobacter typhlonius 1	Epsilonproteobacteria
2721280722	EnvC	WFGY	SS		Ga0174992_111608	2718218393	Chromobacterium vaccinii 21-1	Betaproteobacteria
2774610591	EnvC	WVAY	SS		Ga0226477_111805	2773857813	Pasteurella dagmatis NCTC 11617	Gammaproteobacteria

2776476332	EnvC	LQAY	SS		Ga0263277_112213	2775506949	Mariprofundus ferrinatatus CP-8	Zetaproteobacteria
2804933310	EnvC	-NKY	SS (low)	3XCC	Ga0263390_112575	2802429312	Halobacteriovorax marinus BE01	Deltaproteobacteria
2813736977	EnvC	WTGY	SS	NPV_P10	Ga0325206_112587	2811995294	Acidithiobacillus caldus ATCC 51756	Acidithiobacillia
2832892936	EnvC	WVNY	other		Ga0335562_374	2832892563	Salinimonas sediminis N102	Gammaproteobacteria
2832957764	EnvC	WWGY	SS		Ga0335639_1702	2832956063	Salinisphaera sp. LB1	Gammaproteobacteria
637056001	EnvC	HDHH	SS		PA5551	637000218	Pseudomonas aeruginosa PAO1	Gammaproteobacteria
637773819	EnvC	SDMH	SS	ATG16	Cag_1281	637000072	Chlorobium chlorochromatii CaD3	Chlorobia
648173875	EnvC	VSSF	no TM	LysM	STHERM_c10110	648028053	Spirochaeta thermophila DSM 6192	Spirochaetia
649962061	EnvC	SGVN	SS	2XCC	Odosp_2736	649633078	Odoribacter splanchnicus 1651/6, DSM 20712	Bacteroidia

2813737047	EnvC	HDRH	SS (low)		Ga0325206_112657	2811995294	Acidithiobacillus caldus ATCC 51756	Acidithiobacillia
637054833	LmdC	HDHH	TM	2XCC	PA4404	637000218	Pseudomonas aeruginosa PAO1	Gammaproteobacteria
637087916	LmdC	HDHH	TM	DUF5930+2x CC	CC1872	637000061	Caulobacter vibrioides CB15	Alphaproteobacteria
637182558	LmdC	HDHH	TM	CC	SMc00539	637000269	Sinorhizobium meliloti 1021	Alphaproteobacteria
637475586	LmdC	HDHH	TM	2XCC	RPA2455	637000239	Rhodopseudomonas palustris CGA009	Alphaproteobacteria
637772705	LmdC	HDHH	no TM		Cag_0178	637000072	Chlorobium chlorochromatii CaD3	Chlorobia
638026685	LmdC	HDHH	TM	2xCC	MXAN_5348	637000186	Myxococcus xanthus DK 1622	Deltaproteobacteria
638145187	LmdC	HDHH	no TM	DUF5930+2x CC	HNE_2628	637000135	Hyphomonas neptunium ATCC 15444	Alphaproteobacteria
639297320	LmdC	HDHH	TM	2XCC	Atu1832	639279302	Agrobacterium tumefaciens C58-UWash	Alphaproteobacteria

643383866	LmdC	HDHH	TM	1 CC Nterm, 1 Cterm	HPG27_1481	643348558	Helicobacter pylori G27	Epsilonproteobacteria
643383867	LmdC	EDKH	other	CC	HPG27_1482	643348558	Helicobacter pylori G27	Epsilonproteobacteria
643725271	LmdC	HDHH	TM		NAMH_1536	643692029	Nautilia profundicola Am-H	Epsilonproteobacteria
648147827	LmdC	HDHH	TM	2XCC	Galf_0344	648028028	Gallionella capsiferriformans ES-2	Betaproteobacteria
648174726	LmdC	HDHH	TM		STHERM_c18630	648028053	Spirochaeta thermophila DSM 6192	Spirochaetia
649961209	LmdC	HDHH	TM	2XCC	Odosp_1875	649633078	Odoribacter splanchnicus 1651/6, DSM 20712	Bacteroidia
2508709766	LmdC	HDHH	TM	2XCC	Dsui_0228	2508501046	Azospira oryzae PS	Betaproteobacteria
2510234963	LmdC	HDHH	TM	DUF5930+2x CC	RLO149_c014280	2510065042	Roseobacter litoralis Och 149	Alphaproteobacteria
2510238067	LmdC	HDNH	SS	1 CC C-term	RLO149_p830320	2510065042	Roseobacter litoralis Och 149	Alphaproteobacteria
2511846261	LmdC	HDHH	TM	DUF5930+2x CC	F11_09600	2511231162	Rhodospirillum rubrum F11	Alphaproteobacteria

2540577598	LmdC	HDHH	TM	2-3xCC	LFE_0756	2540341086	Leptospirillum ferrooxidans C2-3	Nitrospira
2563184549	LmdC	HDHH	TM	CC	XALC_2806	2562617136	Xanthomonas albilineans GPE PC73	Gammaproteobacteria
2578483602	LmdC	HDHH	TM	2xCC	EP01_13930	2576861479	Bdellovibrio bacteriovorus 109J	Deltaproteobacteria
2645940631	LmdC	HDHH	(weak) no TM	CC	Ga0100913_11892	2645727549	Vibrio cholerae O1	Gammaproteobacteria
2645942671	LmdC	HDHH	no TM	CC (far N-term)	Ga0100913_121983	2645727549	Vibrio cholerae O1	Gammaproteobacteria
2646276526	LmdC	HDHH	TM	2xCC	Ga0046402_113056	2645727629	Desulfovibrio vulgaris RCH1	Deltaproteobacteria
2668358294	LmdC	HDHH	TM	CC	Ga0110964_111102	2667527488	Campylobacter jejuni CJ677CC008	Epsilonproteobacteria
2668619681	LmdC	HDHH	(weak) no TM	DUF5930+2x CC	Ga0105927_112169	2667527571	Rhodovulum sulfidophilum DSM 1374	Alphaproteobacteria
2776474433	LmdC	HDHH	TM	2XCC	Ga0263277_11312	2775506949	Mariprofundus ferrinatatus CP-8	Zetaproteobacteria
2804932147	LmdC	HDHH	TM	2XCC	Ga0263390_111411	2802429312	Halobacteriovorax marinus BE01	Deltaproteobacteria

2832895534	LmdC	HDHH	no TM (weak)	2XCC	Ga0335562_2972	2832892563	Salinimonas sediminis N102	Gammaproteobacteria
2841602312	LmdC	HDHH	TM	DUF5930+2x CC	Ga0399067_4647	2841597668	Paracoccus denitrificans ATCC 19367	Alphaproteobacteria
2848966019	LmdC	HDHH	TM	2XCC	Ga0349804_01_1850860 _1851837	2848964341	Flavobacterium album HYN0059	Flavobacteriia
637054043	NlpD	NDKH	LIPO	LysM	PA3623	637000218	Pseudomonas aeruginosa PAO1	Gammaproteobacteria
637055365	NlpD	NRKY	LIPO	LysM	PA4924	637000218	Pseudomonas aeruginosa PAO1	Gammaproteobacteria
637088044	NlpD	NNQH	SS	4xLysM	CC1996	637000061	Caulobacter vibrioides CB15	Alphaproteobacteria
637142275	NlpD	NDQH	other	LysM	NGO1056	637000188	Neisseria gonorrhoeae FA 1090	Betaproteobacteria
637182337	NlpD	NNQH	LIPO	2x LysM	SMc02060	637000269	Sinorhizobium meliloti 1021	Alphaproteobacteria
637475976	NlpD	NNQH	LIPO	2xLysM	RPA2837	637000239	Rhodopseudomon as palustris CGA009	Alphaproteobacteria

639297188	NlpD	NNQH	LIPO	2xLysM	Atu1700	639279302	Agrobacterium tumefaciens C58-UWash	Alphaproteobacteria
640674509	NlpD	NDKH	LIPO	LysM	CGSHi22121_01077	640612215	Haemophilus influenzae NTHi 22.1-21, ST176	Gammaproteobacteria
644792400	NlpD	NDKH	LIPO	LysM	Vapar_1799	644736413	Variovorax paradoxus S110	Betaproteobacteria
644792612	NlpD	NDKH	LIPO		Vapar_2015	644736413	Variovorax paradoxus S110	Betaproteobacteria
648148602	NlpD	RDKH	LIPO	LysM	Galf_1120	648028028	Gallionella capsiferriformans ES-2	Betaproteobacteria
2508711508	NlpD	NDKH	LIPO	LysM	Dsui_1970	2508501046	Azospira oryzae PS	Betaproteobacteria
2510236001	NlpD	NNFH	LIPO	LysM	RLO149_c024710	2510065042	Roseobacter litoralis Och 149	Alphaproteobacteria
2511846158	NlpD	NNQH	other	2xLysM	F11_09095	2511231162	Rhodospirillum rubrum F11	Alphaproteobacteria
2563182911	NlpD	RDMH	LIPO	LysM	XALC_1160	2562617136	Xanthomonas albilineans GPE PC73	Gammaproteobacteria

2600369590	NlpD	NDRH	LIPO	LysM	Ga0063390_01052	2600254932	Escherichia coli K-12 MG1655	Gammaproteobacteria
2600369719	NlpD	NDRH	LIPO	LysM	Ga0063390_01181	2600254932	Escherichia coli K-12 MG1655	Gammaproteobacteria
2630388615	NlpD	TSKH	LIPO	LysM	Ga0078022_1197	2627854142	Nitrosomonas communis Nm2	Betaproteobacteria
2645943067	NlpD	NDRH	LIPO	LysM	Ga0100913_122380	2645727549	Vibrio cholerae O1	Gammaproteobacteria
2668620376	NlpD	NDFH	LIPO	LysM	Ga0105927_112865	2667527571	Rhodovulum sulfidophilum DSM 1374	Alphaproteobacteria
2721280365	NlpD	NEKH	LIPO	LysM	Ga0174992_111248	2718218393	Chromobacterium vaccinii 21-1	Betaproteobacteria
2721280713	NlpD	NDMH	LIPO	LysM	Ga0174992_111599	2718218393	Chromobacterium vaccinii 21-1	Betaproteobacteria
2774610438	NlpD	NDKH	LIPO	2xLysM	Ga0226477_111651	2773857813	Pasteurella dagmatis NCTC 11617	Gammaproteobacteria
2812894540	NlpD	NDKH	LIPO	LysM	Ga0213641_111237	2811995033	Comamonas aquatica CJG	Betaproteobacteria
2832893034	NlpD	QEKH	LIPO	LysM	Ga0335562_472	2832892563	Salinimonas sediminis N102	Gammaproteobacteria

2832958965	NlpD	REMH	LIPO	LysM	Ga0335639_2903	2832956063	Salinisphaera sp. LB1	Gammaproteobacteria
2841599160	NlpD	ND-H	LIPO	LysM	Ga0399067_1495	2841597668	Paracoccus denitrificans ATCC 19367	Alphaproteobacteria
638025794	NlpD-HDH	HDHH	LIPO	LysM	MXAN_4449	637000186	Myxococcus xanthus DK 1622	Deltaproteobacteria
649961103	NlpD-HDH	HDHH	LIPO		Odosp_1768	649633078	Odoribacter splanchnicus 1651/6, DSM 20712	Bacteroidia
2578482061	NlpD-HDH	HDHH	LIPO		EP01_06065	2576861479	Bdellovibrio bacteriovorus 109J	Deltaproteobacteria
2646273945	NlpD-HDH	HDHH	no TM	Rod-binding	Ga0046402_11465	2645727629	Desulfovibrio vulgaris RCH1	Deltaproteobacteria
2776475154	NlpD-HDH	HDHH	LIPO	LysM	Ga0263277_111034	2775506949	Mariprofundus ferrinatatus CP-8	Zetaproteobacteria
2804932502	NlpD-HDH	HDHH	LIPO		Ga0263390_111766	2802429312	Halobacteriovorax marinus BE01	Deltaproteobacteria
637088295	LdpB	HDHC	no TM		CC2248	637000061	Caulobacter vibrioides CB15	Alphaproteobacteria
638024079	LdpB	HDHH	SS		MXAN_2713	637000186	Myxococcus xanthus DK 1622	Deltaproteobacteria

640819691	LdpB	HDHH	TM		NIS_0417	640753037	Nitratiruptor sp. SB155-2	Epsilonproteobacteria
643725284	LdpB	HDHH	SS (low)		NAMH_1549	643692029	Nautilia profundicola Am-H	Epsilonproteobacteria
648173518	LdpB	HDHH	SS		STHERM_c06540	648028053	Spirochaeta thermophila DSM 6192	Spirochaetia
2540576950	LdpB	HDVH	SS		LFE_0108	2540341086	Leptospirillum ferrooxidans C2-3	Nitrospira
2540578364	LdpB	HDTF	no TM	CC	LFE_1522	2540341086	Leptospirillum ferrooxidans C2-3	Nitrospira
2563183659	LdpB	HDHH	no TM		XALC_1914	2562617136	Xanthomonas albilineans GPE PC73	Gammaproteobacteria
2646274936	LdpB	HDHH	TM		Ga0046402_111459	2645727629	Desulfovibrio vulgaris RCH1	Deltaproteobacteria
2649937791	LdpB	HDHH	SS (low)		Ga0077940_11433	2648501445	Sulfurovum lithotrophicum ATCC BAA-797	Epsilonproteobacteria
2668357328	LdpB	HDHH	TM	1 CC	Ga0110964_11135	2667527488	Campylobacter jejuni CJ677CC008	Epsilonproteobacteria

2668358441	LdpB	HDHH	SS (low)		Ga0110964_111249	2667527488	Campylobacter jejuni CJ677CC008	Epsilonproteobacteria
2689560212	LdpB	HDHH	SS	2xCC (far N-term)	Ga0133481_111071	2687453639	Helicobacter typhlonius 1	Epsilonproteobacteria
2689561088	LdpB	HDHH	TM		Ga0133481_111949	2687453639	Helicobacter typhlonius 1	Epsilonproteobacteria
2776474743	LdpB	HDHH	SS		Ga0263277_11622	2775506949	Mariprofundus ferrinatatus CP-8	Zetaproteobacteria
2804931770	LdpB	HDHH	LIPO	CC (far N-term)	Ga0263390_111034	2802429312	Halobacteriovorax marinus BE01	Deltaproteobacteria
2832894423	LdpB	HDHD	SS		Ga0335562_1861	2832892563	Salinimonas sediminis N102	Gammaproteobacteria
637054206	LdpB	HDHH	SS	2x LytM	PA3787	637000218	Pseudomonas aeruginosa PAO1	Gammaproteobacteria
644794202	LdpB	HDHH	other	2x LytM	Vapar_3617	644736413	Variovorax paradoxus S110	Betaproteobacteria
2508710896	LdpB	HDHH	SS	2x LytM	Dsui_1358	2508501046	Azospira oryzae PS	Betaproteobacteria
2812894571	LdpB	HDHH	TAT	2x LytM	Ga0213641_111268	2811995033	Comamonas aquatica CJG	Betaproteobacteria

[a] Summary of N-terminal signal sequence prediction from SignalP-5.0: SS indicates a standard secretory signal peptide transported by the Sec translocon and cleaved by signal peptidase I; LIPO indicates a lipoprotein signal peptide transported by the Sec translocon and cleaved

by signal peptidase II; and TAT indicates a twin arginine translocon signal peptide transported by TAT and cleaved by signal peptidase I. TM indicates that in the absence of the aforementioned signals, the sequence was predicted to contain a transmembrane segment by TMHMM. “Low” indicates a predicted signal below the standard threshold. “no TM" indicates that no signal sequence or TM was detected.

[b] Protein domains detected in TREND or coiled-coil domains detected by DeepCoil.

Supplementary Table 2. LytM Clade Member Presence/Absence by Species [a]

Class	Order	Genus	MepM	LmdC	LdpB	NlpD (HDH)	NlpD	EnvC	Other
Acidithiobacillia	Acidithiobacillales	Acidithiobacillus caldus ATCC 51756						2	
Alphaproteobacteria	Caulobacterales	Caulobacter vibrioides CB15	1	1	1		1	1	2
Alphaproteobacteria	Caulobacterales	Hyphomonas neptunium ATCC 15444	2	1				1	2
Alphaproteobacteria	Rhizobiales	Agrobacterium tumefaciens C58-UWash	1	1			1	1	
Alphaproteobacteria	Rhizobiales	Rhodopseudomonas palustris CGA009	1	1			1	1	
Alphaproteobacteria	Rhizobiales	Sinorhizobium meliloti 1021	1	1			1	1	
Alphaproteobacteria	Rhodobacterales	Paracoccus denitrificans ATCC 19367		1			1		
Alphaproteobacteria	Rhodobacterales	Rhodovulum sulfidophilum DSM 1374		1			1		
Alphaproteobacteria	Rhodobacterales	Roseobacter litoralis Och 149		2			1		
Alphaproteobacteria	Rhodospirillales	Rhodospirillum rubrum F11	2	1			1	1	
Bacteroides-Flavobacteria	Bacteroidales	Odoribacter splanchnicus 1651/6, DSM 20712	1	1		1		1	1
Betaproteobacteria	Burkholderiales	Comamonas aquatica CJG	1		1		1		
Betaproteobacteria	Burkholderiales	Variovorax paradoxus S110	1		1		2		
Betaproteobacteria	Neisseriales	Chromobacterium vaccinii 21-1	1				2	1	
Betaproteobacteria	Neisseriales	Neisseria gonorrhoeae FA 1090	1				1	1	
Betaproteobacteria	Neisseriales	Gallionella capsiferriformans ES-2	1	1			1	1	
Betaproteobacteria	Nitrosomonadales	Nitrosomonas communis Nm2	1				1	1	
Betaproteobacteria	Rhodocyclales	Azospira oryzae PS	1	1	1		1	1	
Chlorobia	Chlorobiales	Chlorobium chlorochromatii CaD3		1				1	
Deltaproteobacteria	Bacteriovoracales	Halobacteriovorax marinus BE01	1	1	1	1		1	1
Deltaproteobacteria	Bdellovibrionales	Bdellovibrio bacteriovorus 109J		1		1		1	1
Deltaproteobacteria	Desulfovibrionales	Desulfovibrio vulgaris RCH1	1	1	1	1		1	
Deltaproteobacteria	Myxococcales	Myxococcus xanthus DK 1622	1	1	1	1		1	4

Epsilonproteobacteria	Campylobacterales	Campylobacter jejuni CJ677CC008	1	1	2			1	
Epsilonproteobacteria	Campylobacterales	Helicobacter pylori G27	1	2				1	
Epsilonproteobacteria	Campylobacterales	Helicobacter typhlonius 1	1		2			1	
Epsilonproteobacteria	Nautiliales	Nautilia profundicola Am-H	1	1	1			1	
Epsilonproteobacteria	unclassified	Nitratiruptor sp. SB155-2	1		1			1	
Epsilonproteobacteria	unclassified	Sulfurovum lithotrophicum ATCC BAA-797	1		1			1	
Bacteroides-Flavobacteria	Flavobacteriales	Flavobacterium album HYN0059	1	1					1
Gammaproteobacteria	Alteromonadales	Salinimonas sediminis N102	1	1	1		1	1	1
Gammaproteobacteria	Enterobacterales	Escherichia coli K-12 MG1655	1				2	1	
Gammaproteobacteria	Pasteurellales	Haemophilus influenzae NTHi 22.1-21, ST176	1				1	1	
Gammaproteobacteria	Pasteurellales	Pasteurella dagmatis NCTC 11617	1				1	1	
Gammaproteobacteria	Pseudomonadales	Pseudomonas aeruginosa PAO1	1	1	1		2	1	1
Gammaproteobacteria	Salinisphaerales	Salinisphaera sp. LB1	3				1	1	
Gammaproteobacteria	Vibrionales	Vibrio cholerae O1	2	2			1	1	
Gammaproteobacteria	Xanthomonadales	Xanthomonas albilineans GPE PC73	1	1	1		1	1	1
Nitrospira	Nitrospirales	Leptospirillum ferrooxidans C2-3		1	2				1
Spirochaetia	Spirochaetales	Spirochaeta thermophila DSM 6192		1	1			1	3
Zetaproteobacteria	Mariprofundales	Mariprofundus ferrinatatus CP-8	1	1	1	1		1	

[a] Numbers indicate identified genes from each clade; some species had multiple paralogs of certain LytM factors. The "Other" category clusters non-related genes that did not clearly segment into a large clade.

Supplementary Table 3: Bacterial strains and plasmids used in this study

Strain or plasmid	Relevant characteristics	Reference/Source
pTNS3	Ap^r; helper plasmid encoding the site-specific TnsABCD Tn7 transposition pathway	(Choi KH, et al., 2008)
pUC18-mini-Tn7T-GM-Ptac-sf*gfp*	Ap^r Gm^r; mini-Tn7 vector containing sfGFP under control of the *tac* promoter	(Figueroa-Cuilan et al., 2016)
pUC18-mini-Tn7T-GM-Ptac-*Atu1700*$_{STOP,}$-sf*gfp*	Ap^r Gm^r; mini-Tn7 vector containing *dipM*$_{STOP}$ under control of the *tac* promoter	This study
pUC18-mini-Tn7T-GM-Plac-sf*gfp*	Ap^r Gm^r; mini-Tn7 vector containing sfGFP under control of the *lac* promoter	(Figueroa-Cuilan et al., 2016)
pUC18-mini-Tn7T-GM-Plac-*Atu4178*$_{STOP,}$-sf*gfp*	Ap^r Gm^r; mini-Tn7 vector containing *rgsM*$_{STOP, ATU}$ under control of the *tac* promoter	This study
pNTPS139	Km^r; Suicide vector containing *oriT* and *sacB*	D. Alley
pNTPS139 *ΔAtu1700*	Kmr Sucs; deletion plasmid for *dipM*$_{ATU}$	This study
pNTPS139 *ΔAtu2275*	Kmr Sucs; deletion plasmid for *envC*$_{ATU}$	This study
pNTPS139 *ΔAtu4178*	Kmr Sucs; deletion plasmid for *rgsM*$_{ATU}$	This study
pNTPS139 *ΔAtu1340*	Kmr Sucs; deletion plasmid for *amiC*$_{ATU}$	This study
pNTPS139 *ΔAtu2113*	Kmr Sucs; deletion plasmid for *ampD*$_{ATU}$	(Figueroa-Cuilan et al., 2020)
pSRKKm-Plac	Km^r; pSRKKm vector containing lacI^q and *lac* promoter	(Khan et al., 2008)
pSRKKm-Plac-sf*gfp*	Km^r; pSRKKm vector containing lacI^q and *lac*	(Figueroa-Cuilan et al., 2016)

	promoter with sfGFP	
pSRKKm-Pcym-*Atu1832*-sf*gfp*	Km^r; pSRKKm vector containing CuO^q and *cym* promoter producing LmdC-sfGFP	This study
pSRKKm-Ptac-sf*gfp*	Km^r; pSRKKm vector containing $lacI^q$ and *tac* promoter with sfGFP	(Figueroa-Cuilan et al., 2016)
pSRKKm-Ptac-$Atu1700_{STOP}$-sf*gfp*	Km^r; pSRKKm vector containing $lacI^q$ and *tac* promoter for the overproduction of DipM	This study
pUC18-mini-Tn7T-GM-Plac-sf*gfp*	Ap^r Gm^r; mini-Tn7 vector containing sfGFP under control of the *lac* promoter	(Figueroa-Cuilan et al., 2016)
pUC18-mini-Tn7T-GM-Plac-$Atu4178_{STOP}$-sf*gfp*	Ap^r Gm^r; mini-Tn7 vector containing $rgsM_{STOP, ATU}$ under control of the *tac* promoter	This study
pSRKKm-T7medium-sf*gfp*	Km^r; pSRKKm vector containing T7 promoter medium expression	This study
pSRKKm-T7medium-*Atu1340*-sf*gfp*	Km^r; pSRKKm vector expressing *amiC* under the T7 medium constitutive control	This study
pSRKKm-T7medium-*Atu2113*-sf*gfp*	Km^r; pSRKKm vector expressing *amiD* under the T7 medium constitutive control	This study
pSRKKm-T7strong-sf*gfp*	Km^r; pSRKKm vector containing T7 strong promoter	This study
pSRKKm-T7strong-*Atu1700*-sf*gfp*	Km^r; pSRKKm vector expressing *dipM* under the T7 strong constitutive control	This study
pSRKKm-Pcym-sf*gfp*	Km^r; pSRKKm vector	This study

	containing *lac* promoter flanked by cuO operator site along with the cymR repressor gene	
pSRKKm-Pcym-*Atu4178*-sf*gfp*	Km^r; pSRKKm vector containing lac promoter flanked by *cuO* operator site along with the cymR repressor gene expressing $rgsM_{ATU}$	This study
pSRKKm-PenvC-sf*gfp*	Km^r; pSRKKm vector containing the EnvC promoter	This study
pSRKKm-PenvC-*Atu2775*-sf*gfp*	Km^r; pSRKKm vector containing the *envC* promoter and producing EnvC under the native control	This study
pMCS-2	Km^r	(Thanbichler et al. 2007)
pMCS-2-*Atu1832*-IKO	Km^r; pMCS-2 vector containing nt 1172-1771 of *Atu1832*	This study
E. coli strains		
DH5α	Cloning strain	Life Technologies
S17-1	Sm^r; RP4-2, Tc::Mu, Km-Tn7, for plasmid mobilization	(Simon et al., 1983)
S17-1 pNTPS139 *ΔAtu1700*	Sm^r; RP4-2, Tc::Mu, Km-Tn7, for mobilizing the *dipM* deletion plasmid	This study
S17-1 pNTPS139 *ΔAtu2775*	Sm^r; RP4-2, Tc::Mu, Km-Tn7, for mobilizing the $envC_{ATU}$ deletion plasmid	This study
S17-1 pNTPS139 *ΔAtu4178*	Sm^r; RP4-2, Tc::Mu, Km-Tn7, for mobilizing the $rgsM_{ATU}$ deletion plasmid	This study

S17-1 pNTPS139 *ΔAtu1340*	Sm^r; RP4-2, Tc::Mu, Km-Tn7, for mobilizing the *amiC*$_{ATU}$ deletion plasmid	This study
S17-1 pNTPS139 *ΔAtu2113*	Sm^r; RP4-2, Tc::Mu, Km-Tn7, for mobilizing the *amiD*$_{ATU}$ deletion plasmid	(Figueroa-Cuilan et al., 2020)
A. tumefaciens strains		
C58Δ*tetRA*::a-*att*Tn7	Parent strain used in this study. *tetRA* locus was replaced with an artificial *att*Tn7 site. Referred to as WT in this study.	(Figueroa-Cuilan et al., 2016)
C58Δ*tetRA*::a-*att*Tn7 pSRKKm-Plac-sf*gfp*	Km^r; C58Δ*tetRA*::a-*att*Tn7 with pSRKm producing sfGFP under the control of the *lac* promoter	(Figueroa-Cuilan et al., 2016)
C58Δ*tetRA*::a-*att*Tn7 pSRKKm-Ptac-sf*gfp*	Km^r; C58Δ*tetRA* ::a-*att*Tn7 with pSRKm producing sfGFP under the control of the *tac* promoter	(Figueroa-Cuilan et al., 2016)
C58Δ*tetRA*::a-*att*Tn7 pSRKKm-Pcym-*Atu1832*-sf*gfp*	Km^r; C58Δ*tetRA*::a-*att*Tn7 with pSRKm producing LmdC-sfGFP under the control of the *cym* promoter	This study
C58Δ*tetRA*::a-*att*Tn7 pSRKKm-PT7strong-sf*gfp*	Km^r; C58Δ*tetRA*::a-*att*Tn7 with pSRKm containing the *T7* strong promoter	This study
C58Δ*tetRA*::a-*att*Tn7 pSRKKm-PT7strong-*Atu1700*-sf*gfp*	Km^r; C58Δ*tetRA*::a-*att*Tn7 with pSRKm constitutively producing DipM-sfGFP under the control of the *T7* strong promoter	This study
C58Δ*tetRA*::a-*att*Tn7 pSRKKm-PenvC-sf*gfp*	Km^r; C58Δ*tetRA*::a-*att*Tn7 with pSRKm containing the native *envC* promoter	This study
C58Δ*tetRA*::a-*att*Tn7 pSRKKm-PenvC-*Atu2775*-sf*gfp*	Km^r; C58Δ*tetRA*::a-*att*Tn7 with pSRKm producing EnvC-sfGFP under the control of the *envC* native	This study

	promoter	
C58Δ*tetRA*::a-*att*Tn7 pSRKKm-Pcym-sf*gfp*	Km^r; C58Δ*tetRA*::a-*att*Tn7 with pSRKm containing the *lac* promoter flanked by *cuO* operator site along with the *cymR* repressor gene	This study
C58Δ*tetRA*::a-*att*Tn7 pSRKKm-Pcym-*Atu4178*-sf*gfp*	Km^r; C58Δ*tetRA*::a-*att*Tn7 with pSRKm producing RgsM-sfGFP under the control of the *cym* promoter	This study
C58Δ*tetRA*::a-*att*Tn7 pSRKKm-Ptac-*Atu1700*$_{STOP}$-sf*gfp*	Km^r; C58Δ*tetRA*::a-*att*Tn7 with pSRKm overproducing DipM under the control of the *tac* promoter	This study
C58Δ*tetRA*::mini-Tn7T-GM-lac-sf*gfp*	Gm^r; C58Δ*tetRA*::a-*att*Tn7 with mini Tn7 producing sfGFP under the control of the *lac* promoter	(Figueroa-Cuilan et al., 2016)
C58Δ*tetRA*::mini-Tn7T-GM-lac-*Atu4178*$_{STOP}$-sf*gfp*	Gm^r; C58Δ*tetRA*::a-*att*Tn7 with mini Tn7 producing RgsM$_{STOP, ATU}$ under the control of the *lac* promoter	This study
C58Δ*tetRA*::a-*att*Tn7 *Atu1832::*pMCS-2-*Atu1832*-IKO	Km^r; C58Δ*tetRA*::a-*att*Tn7 with insertional knockout of *lmdC*$_{ATU}$	This study
C58Δ*tetRA*::mini-Tn7T-GM-lac-*Atu4178*$_{STOP}$-sf*gfp ΔAtu4178*	Gm^r; C58Δ*tetRA*::a-*att*Tn7 Δ*rgsM* with mini Tn7 expressing *rgsM*$_{STOP, ATU}$ under the control of the *lac* promoter	This study
C58Δ*tetRA*::mini-Tn7T-GM-tac-*Atu1700*$_{STOP}$-sf*gfp ΔAtu1700* pSRKKm-Plac-sf*gfp*	Gm^r, Km^r; C58Δ*tetRA*::a-*att*Tn7 *ΔdipM* expressing *dipM*$_{STOP, ATU}$ under the control of the *tac* promoter on the Mini-Tn7 system. DipM depletion strain. Producing extra *lac* repressor to prevent	This study

	promoter leakiness.	
C58Δ*tetRA*::a-*att*Tn7 *ΔAtu2775*	Deletion of $envC_{ATU}$ in C58Δ*tetRA*::a-*att*Tn7	This study
C58Δ*tetRA*::a-*att*Tn7 *ΔAtu1340*	Deletion of $amiC_{ATU}$ in C58Δ*tetRA*::a-*att*Tn7	This study
C58Δ*tetRA*::a-*att*Tn7 *ΔAtu2113*	Deletion of $amiD_{ATU}$ in C58Δ*tetRA*::a-*att*Tn7	(Figueroa-Cuilan et al., 2020)
C58Δ*tetRA*::a-*att*Tn7 *ΔAtu1340ΔAtu2113*	Deletion of $amiC_{ATU}$ and $amiD_{ATU}$ in C58Δ*tetRA*::a-*att*Tn7	This study
C58Δ*tetRA*::a-*att*Tn7 *ΔAtu2775ΔAtu1340*	Deletion of $amiC_{ATU}$ and $envC_{ATU}$ in C58Δ*tetRA*::a-*att*Tn7	This study
C58Δ*tetRA*::a-*att*Tn7 *ΔAtu2775* pSRKKm-PenvC-sf*gfp*	Km^r; Deletion of $envC_{ATU}$ in C58Δ*tetRA*::a-*att*Tn7 with empty vector	This study
C58Δ*tetRA*::a-*att*Tn7 *ΔAtu2775* pSRKKm-PenvC-*Atu2775*-sf*gfp*	Km^r; Deletion of $envC_{ATU}$ in C58Δ*tetRA*::a-*att*Tn7 carrying complementing plasmid	This study
C58Δ*tetRA*::a-*att*Tn7 *ΔAtu1340* pSRKKm-PT7 medium-sf*gfp*	Km^r; Deletion of $amiC_{ATU}$ in C58Δ*tetRA*::a-*att*Tn7	This study
C58Δ*tetRA*::a-*att*Tn7 *ΔAtu1340* pSRKKm-PT7medium-*Atu1340* sf*gfp*	Km^r; Deletion of $amiC_{ATU}$ in C58Δ*tetRA*::a-*att*Tn7 carrying complementing plasmid	This study
C58Δ*tetRA*::a-*att*Tn7 Δ*Atu1340* pSRKKm-Ptac-sf*gfp*	Km^r; Deletion of $amiC_{ATU}$ in C58Δ*tetRA*::a-*att*Tn7 with pSRKm containing the *tac* promoter	This study
C58Δ*tetRA*::a-*att*Tn7 Δ*Atu1340* pSRKKm-Ptac-$dipM_{STOP}$-sf*gfp*	Km^r; Overexpression of *dipM* in deletion of $amiC_{ATU}$	This study
C58Δ*tetRA*::a-*att*Tn7 Δ*Atu2113* pSRKKm-Ptac-sf*gfp*	Km^r; Deletion of $amiD_{ATU}$ in C58Δ*tetRA*::a-*att*Tn7 with pSRKm containing the *tac* promoter pSRK empty plasmic	This study

C58Δ*tetRA*a::*att*Tn7 Δ*Atu2113* pSRKKm-Ptac-*Atu1700*$_{STOP}$-sf*gfp*	Km^r; Overexpression of *dipM* in deletion of *amiC*$_{ATU}$	This study
C58Δ*tetRA*::a-*att*Tn7 Δ*Atu1340*Δ*Atu2113* pSRKKm-Ptac-sf*gfp*	Km^r; Double deletion of *amiC*$_{ATU}$ and *amiD*$_{ATU}$ in C58Δ*tetRA*::a-*att*Tn7 with pSRKm containing the *tac* promoter	This study
C58Δ*tetRA*:a-*att*Tn7 Δ*Atu1340*Δ*Atu2113* pSRKKm-Ptac-*Atu1700*$_{STOP}$-sf*gfp*	Km^r; Overexpression of *dipM* in the double deletion of *amiC*$_{ATU}$ and *amiD*$_{ATU}$	This study

*ATU = *Agrobacterium tumefacies* (C58) gene

Supplementary Table 4

Synthesized DNA primers used in this study

**Synthesized DNA**	**Sequence**
**Primers**	
T7forwardEcoRINdeI	5'-AAT TCT AAT ACG ACT CAC TAT AGG GAG ACA-3'
T7revEcoRINdeI	5'-TATGTCTCCCTATAGTGAGTCGTATTAG-3'
Atu1832 pSRK F	5'-TCGAGTCAAGGAAGACAGCATATGACTGATACGGCGGAAAATC-3'
Atu1832-GFPpSRK_R	5-AACCAGCAGCGGAGCCAGCGGATCCTTTTAGATAGGTTTTCAGCTCC-3'
Atu2775 Ndel F	5'-GTCGTCCATATGGATACTGAGCCT-3'
Atu2775 BamHI R	5'-GTCGTCGGATCCTTACGAATCATTGTGCGC-3'
Atu2775 BamHI (-STOP) R	5'-GTCGTCGGATCCCGAATCATTGTGCGC-3'
PenvC EcoRI F	5'-GAATTCACGGGTTTCGATTCC-3'
Atu2775 AgeI (-STOP) R	5'-GTCGTCACCGGTCGAATCATTGTGCGCCCT-3'
Atu1700 NdeI F	5'-GCACGCCATATGGGCCTTGCAAGAAGTGAAATT-3'
Atu1700 BamHI (-STOP) R	5'-GTCGCTGGATCCTTCAAGGAAACCTGAT-3'
Atu1700 BamHI R	5'GTCGTCGGATCCCTATTCAAGGAAACCTGATGG-3'
Atu4178 Nde1 F	5'-GCGGCGCATATGACTGCGGATCGCAAT-3'
Atu4178 BamH1 (-STOP) R	5'GCGGCGGGATCCGCGGCTCGCCACCTG-3'
Atu1340 NdeI F	5'-GTCGTCCATATGGATTTCACGCGCAGC-3'
Atu1340 BamHI (-STOP) R	5'-GTCGTCGGATCCGCCGCCATTCGC-3'
Atu2113 NdeI F	5'-GTCGTCCATATGAAAGAATGTCTGCCGGAT-3'

Atu2113 BamHI R	5'-GTCGTCGGATCCGGCGGCCGAAAATCT-3'
Atu1340 SpeI P1	5'-GTCGTCACTAGTAGGCGCTTGGCCTGCTCCTGGTAT-3'
Atu1340 P2	5'-AAGCTTGGTACCGAATTCGCGCGTGAAATCCATGGCCCT-3'
Atu1340 P3	5'-GAATTCGGTACCAAGCTTGCCCTGGCGAATGGCGGCTGA-3'
Atu1340 P4	5'-GTCGTCGGATCCATTCTGCCATCAGAGCACCGTTGC-3'
Atu1340 P5	5'-GTCGTCCGATCCGGTTCCCACGGA-3'
Atu1340 P6	5'-TGGAAGCGCCGACCGGGC-3'
Atu1832 IKO pMCS F	5'-TAAGATCTCGAGCTCCGGAGAATTCCTCATCTTGCGCGACGACC-3'
Atu1832 IKO pMCS R	5-ACCGGTACGCGTAACGTTCGAATTCGCCGACATCGTCGGTGCC-3'
Atu2775 SpeI P1	5'-GTCGTCACTAGTGTGCTTCCAATGCCGAGACCCGCA-3'
Atu2775 P2	5'-AAGCTTGGTACCGAATTCTTGAGGCTCAGTATCCATTTGAAC-3'
Atu2775 P3	5'-GAATTCGGTACCAAGCTTGGAAGGGCGCACATTGATTCGTAA-3'
Atu2775 BamHI P4	5'-GTCGTCGGATCCCACCCTTGCGGATCACCGTCAGCT-3'
Atu2775 P5	5'-CCGCAGGCTATGCTGAATAGAGCG-3'
Atu2775 P6	5'-CAGGTCGAGGACATAGCCCTTCAG-3
Atu1700 SpeI P1	5'-GCTAGACTAGTTCCACCGCCGCCTTTAC-3'
Atu1700 P2	5'-AAGCTTGGTACCGAATTCAGACGAATGT-3'
Atu1700 P3	5'-GAATTCGGTACCAAGCTTGGTTTCCTTGA-3'

Atu1700 BamHI P4	5'-GCATAGGGATCCAAGCACGCCCATCATG-3'
Atu1700 P5	5'-TTGAGATCGGCACCGGCAGCGGTTTTA-3'
Atu1700 P6	5'-ATCGTCACCGAATTGACGGCGCGCGGCT-3'
Atu4178 SpeI P1	5'-GTCGTCACTAGTAAAATCAAACCCCAAAAGCTAAA-3'
Atu4178 P2	5'-AAGCTTGGTACCGAATTCGATCCGCAGTCATACTAGC-3'
Atu4178 P3	5'-GAATTCGGTACCAAGCCTATCAGGTGGCGAGCCGCTGA-3'
Atu4178 BamHI P4	5'-GTCGTCGGATCCACAACGACGATGTGGATTCGGTTCGCG-3'
Atu4178 P5	5'-TGCTCGCGAAATTGGTGTTGTCTCATTAA-3'
Atu4178 P6	5'-CCTATCAGGTCGTGTTGTTCGAT-3'

*R = Reverse, F=Forward primer
**AtuXXXX- Represents *Agrobacterium tumefaciens* gene loci
***P1-P4 primers amplify the 500 upstream and downstream regions of the gene loci, whereas P5 and P6 are used to screen for the gene deletion
*****-STOP = Reverse (R) primer does not contain a stop

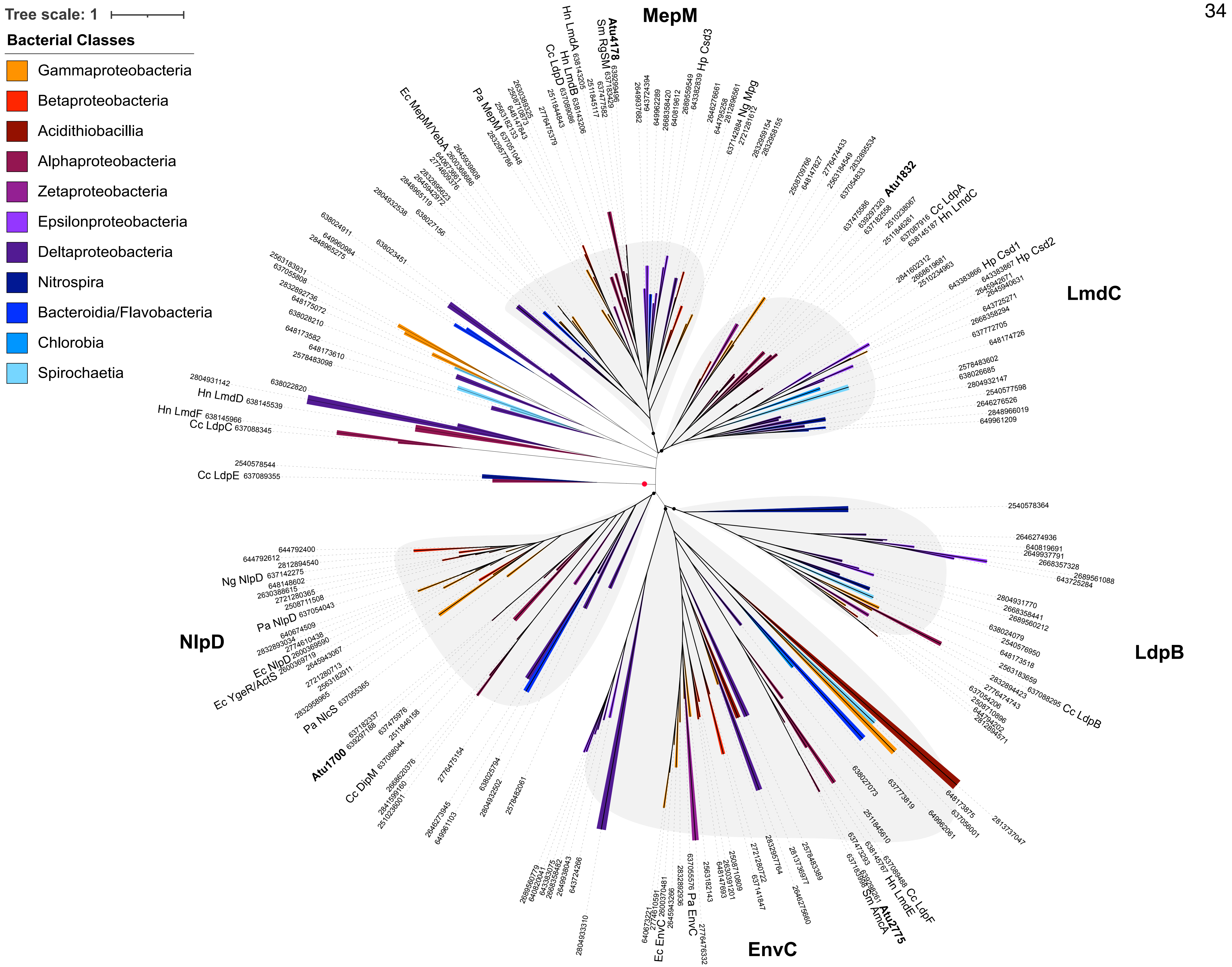

Supplementary Figure 1. Unrooted version of the tree in **Figure 1.** Solid black circles indicate the base of each of the five clades, the boundaries of which are outlined in gray shapes and labeled around the periphery. The solid red circle indicates where the root was placed to create **Figure 1**. The root was chosen to best indicate the clades which are also distinguished by shared N-terminal genetic architecture that was not used to construct this tree.

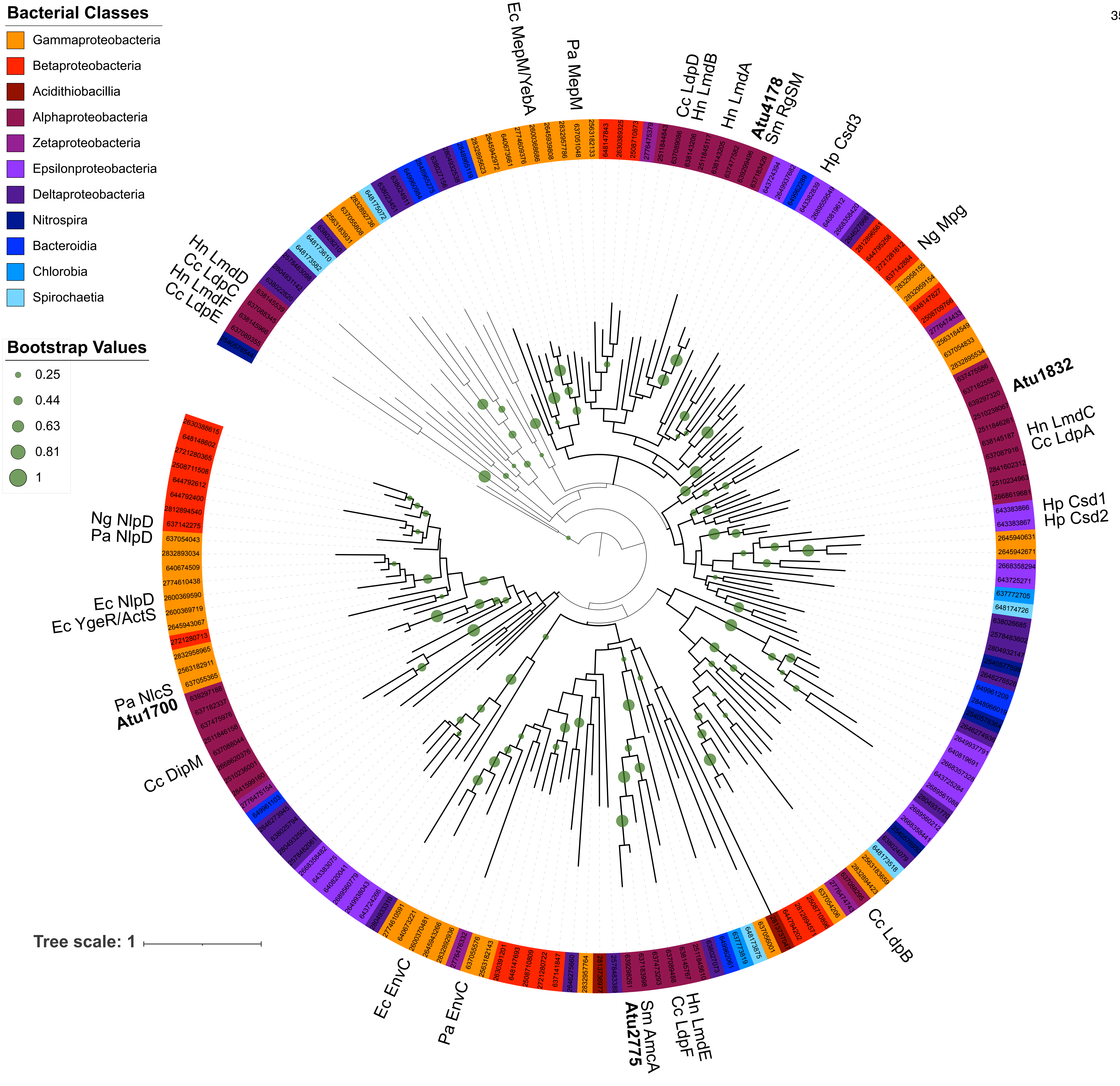

Supplementary Figure 2. The maximum likelihood tree from **Figure 1** showing branch lengths and bootstrap values.

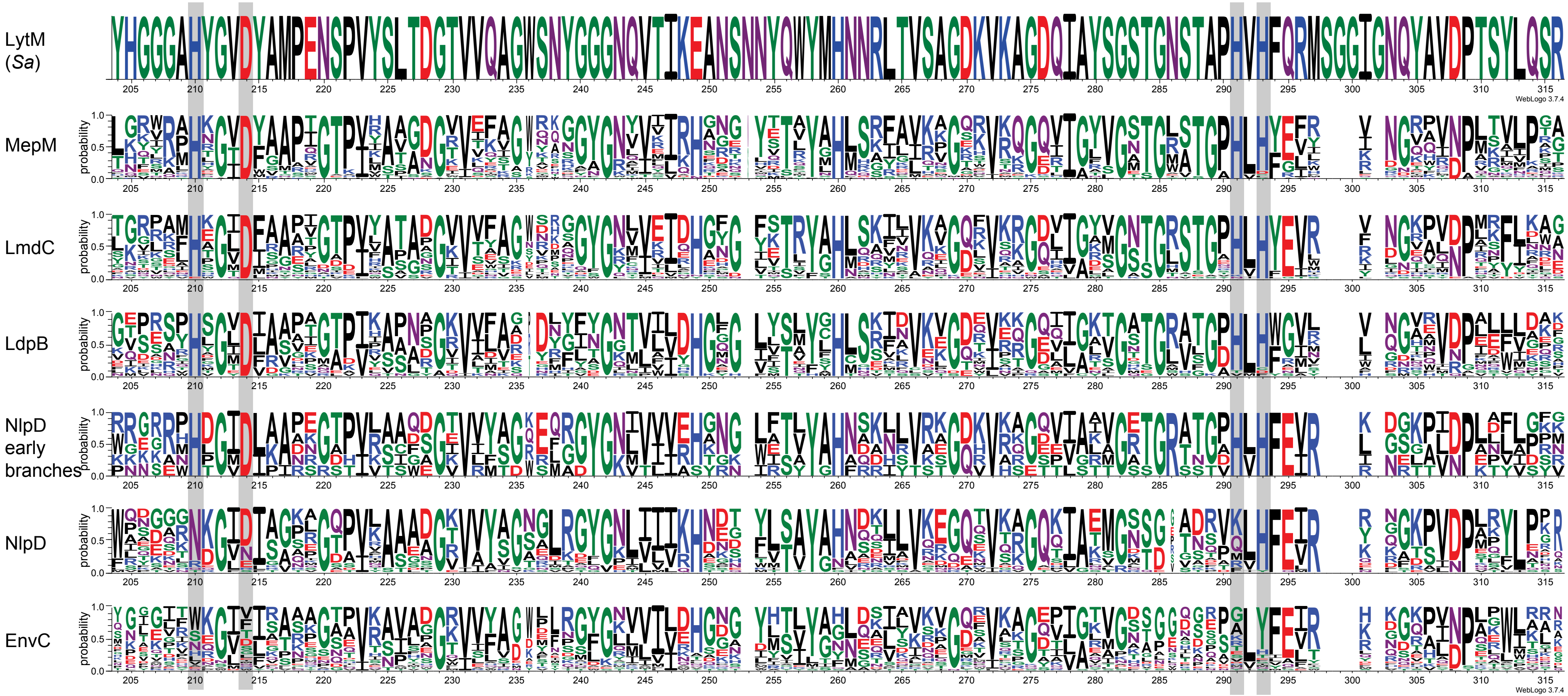

Supplementary Figure 3. HMM logos of LytM domain clades made with WebLogo 3. Logos were constructed from protein sequences of clade members indicated in the gene tree in **Figure 1** and in **Supplementary Table 3**. Amino acids are color coded according to chemical properties, with uncharged polar residues in green, neutral residues in purple, basic residues in blue, acidic residues in red, and hydrophobic residues in black. The height of each letter is propor- tional to the relative frequency of a given identity, and the height of the stack indicates the sequence conservation at that position. Staphylococcus aureus LytM numbering is used for ease of comparison. Gray boxes mark conserved positions in the metalloprotease active site for zinc metal coordination.

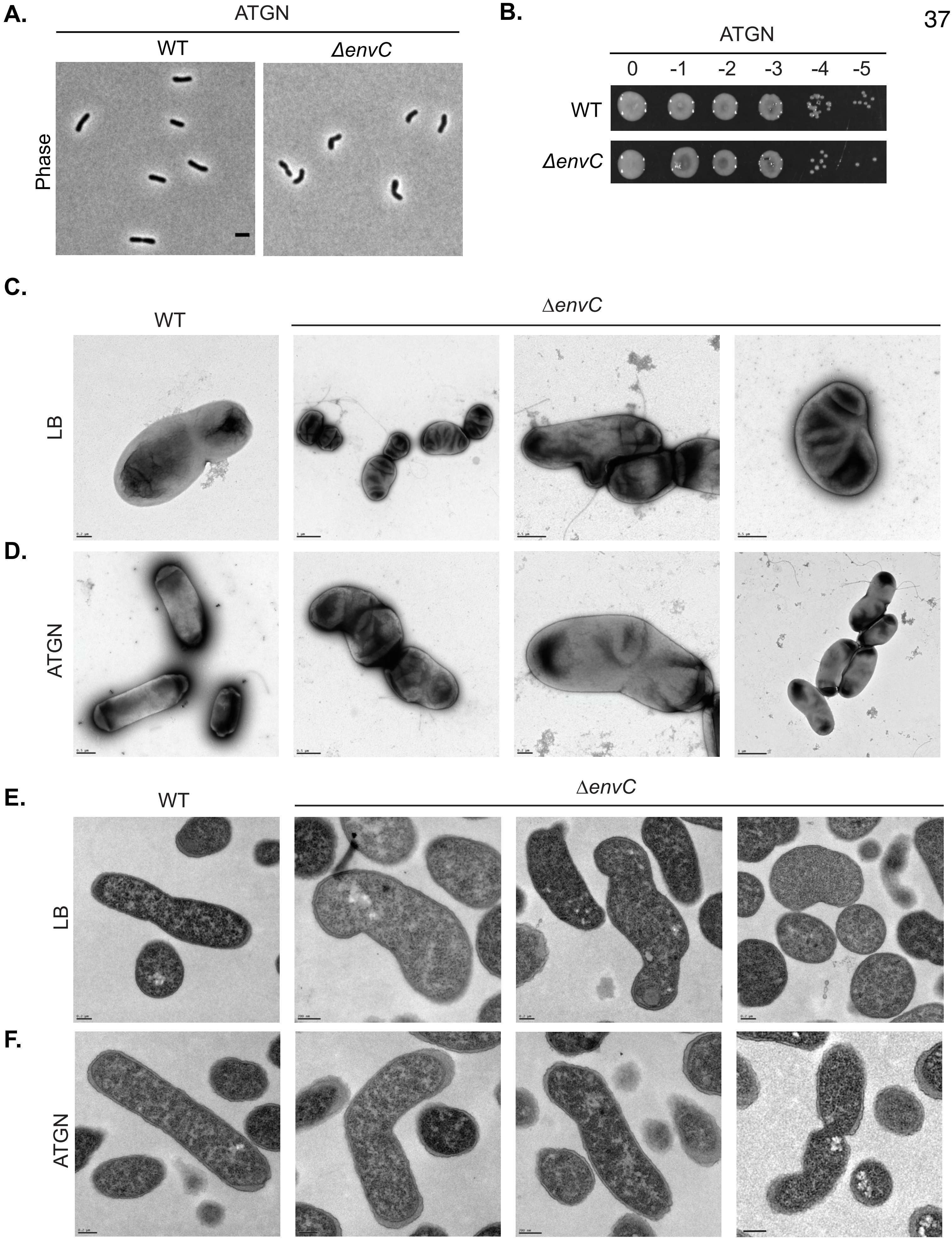

Supplementary Figure 4. Hypercurvature, bending, and kinking of *ΔenvC* cells persists in ATGN medium. (A) Phase contrast images of WT and *ΔenvC* cells grown in ATGN. Scale bar = 2 µm. (B) Viability spot assays of WT and *ΔenvC* cells grown in ATGN. (C-D) Whole cell transmission electron microscopy of WT and *ΔenvC* cells grown in LB (C) and ATGN (D). Note, scale bars are not uniform and are (moving right to left and top to bottom) 0.2 µm, 1 µm, 0.5 µm, 0.5 µm, 0.5 µm, 0.5 µm, 0.2 µm, and 1 µm. (E-F) Thin section transmission electron microscopy of WT and *ΔenvC* cells grown in LB (E) and ATGN (F). Scale bars are 0.2 µm for all images in (E) and (F).

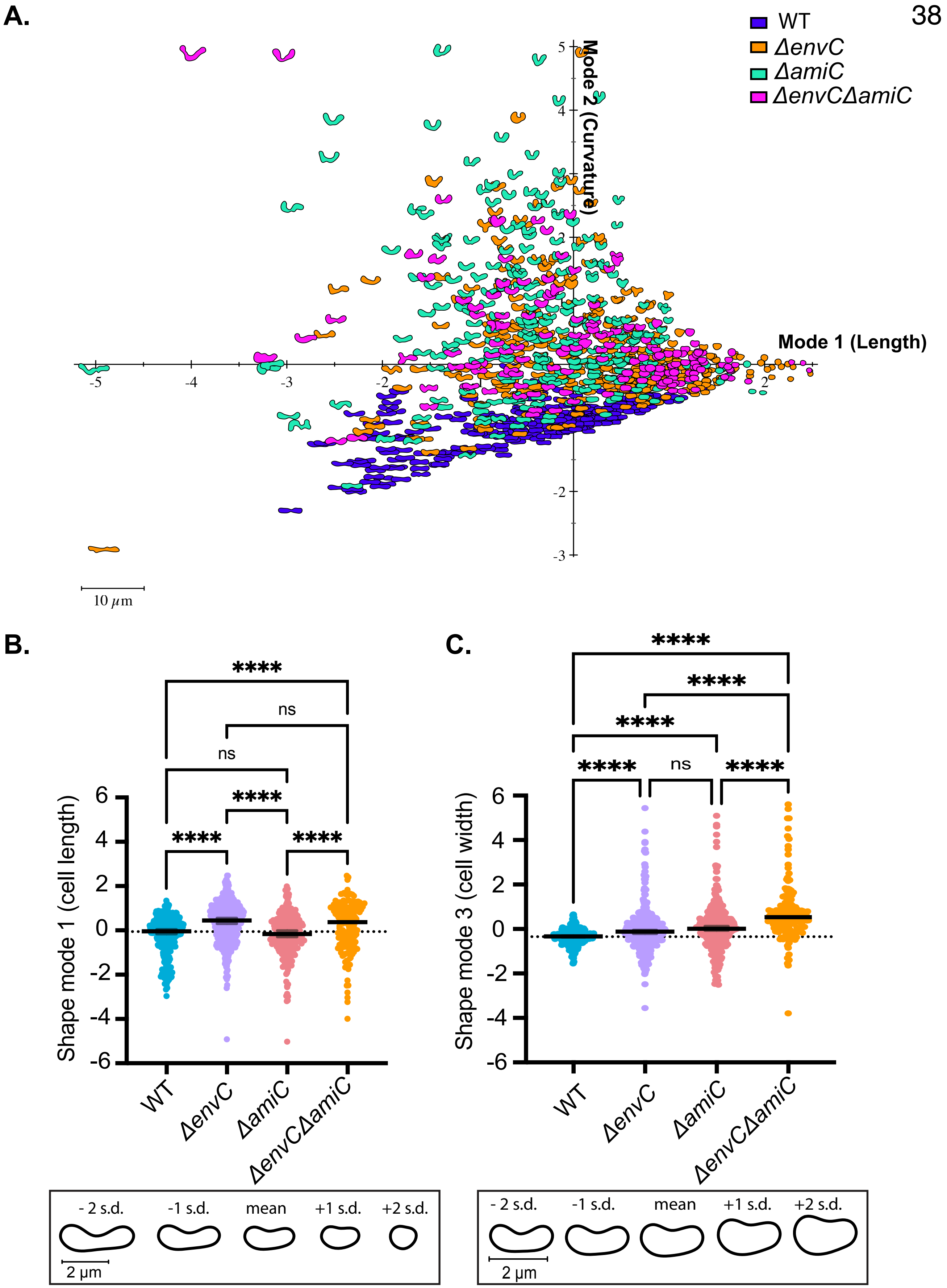

Supplementary Figure 5. Quantitative characterization of cell shape WT, *ΔenvC, ΔamiC* and *ΔenvCΔamiC*. (A) Principal-component analysis (PCA) of cell shape for WT, *ΔenvC, ΔamiC* and *ΔenvCΔamiC*. Shape modes 1, 2 and 3 approximately capture cell length, curvature, and width, respectively. Shape modes 1 (B) and 2 (C) values were plotted. Error bars represent mean ± SEM. The black dashed line represents the WT mean. A Kruskal-Wallis test with Dunn's posttest was used to compare the means of shape mode 1 and a One Way ANOVA with a Tukey posttest was used to compare the means of shape mode 3. ****, P ≤ 0.0001, ns = not significant.

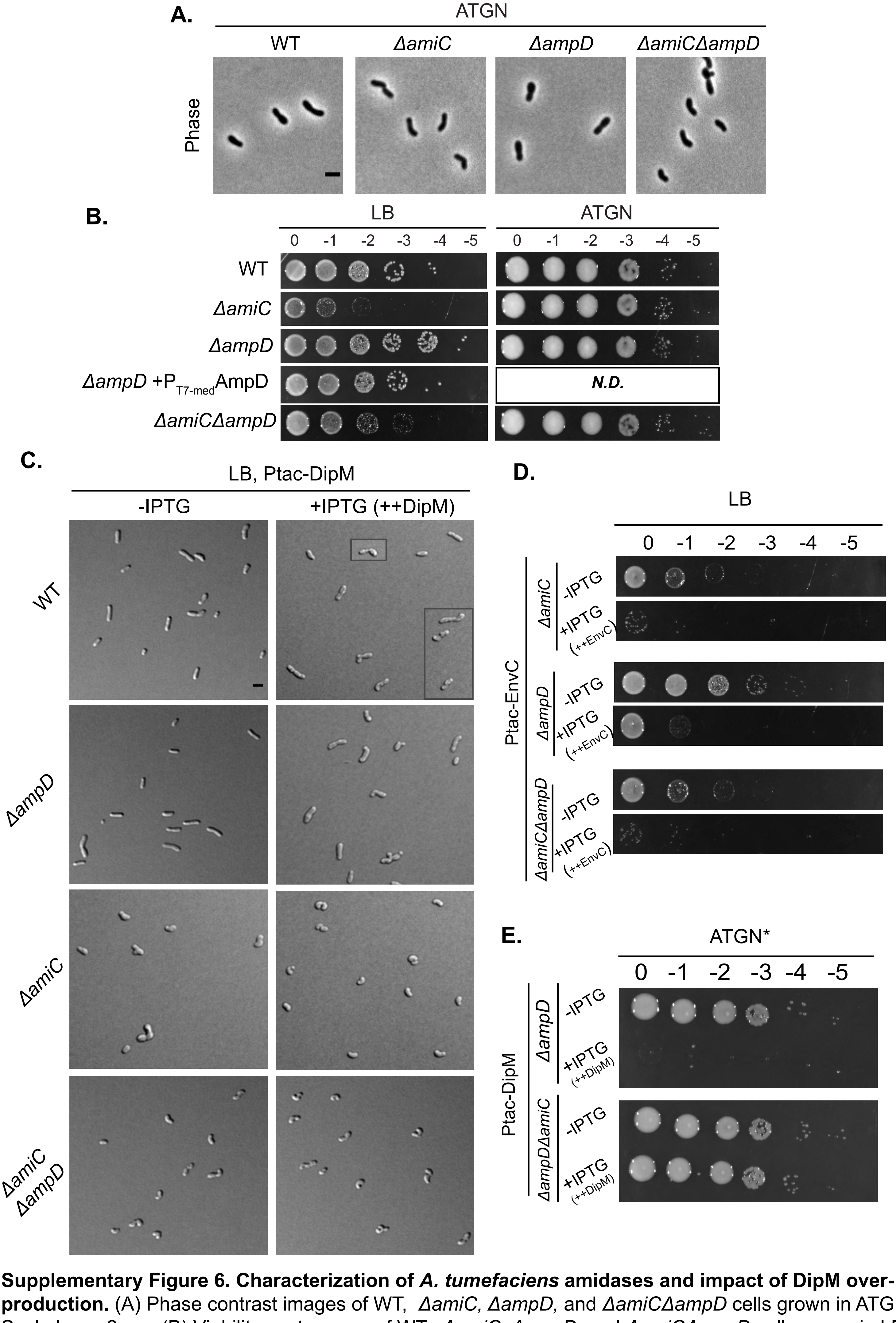

Supplementary Figure 6. Characterization of *A. tumefaciens* amidases and impact of DipM over-production. (A) Phase contrast images of WT, *ΔamiC, ΔampD,* and *ΔamiCΔampD* cells grown in ATGN. Scale bar = 2 μm. (B) Viability spot assays of WT, *ΔamiC, ΔampD*, and *ΔamiCΔampD* cells grown in LB and ATGN. (C) DIC images of WT, *ΔamiC, ΔampD,* and *ΔamiCΔampD* cells grown LB in the absence (left) or presence (right) of DipM overproduction (++DipM). Scale bar = 2 nm. (D) Cell viability *ΔamiC, ΔampD, ΔamiCΔampD* cells containing empty plasmid (P_{tac}-Empty) or overproducing EnvC (+IPTG, ++EnvC). (E) Viability spot assays of *ΔampD* and *ΔamiCΔampD* cells grown in ATGN in the absence (++DipM) or presence of the inducer IPTG.++DipM indicates that DipM is being overproduced. *Since *ΔamiC* has a strong viability defect in LB, the DipM overproduction data was presented in ATGN for better assesment of the DipM overproduction phenotype.

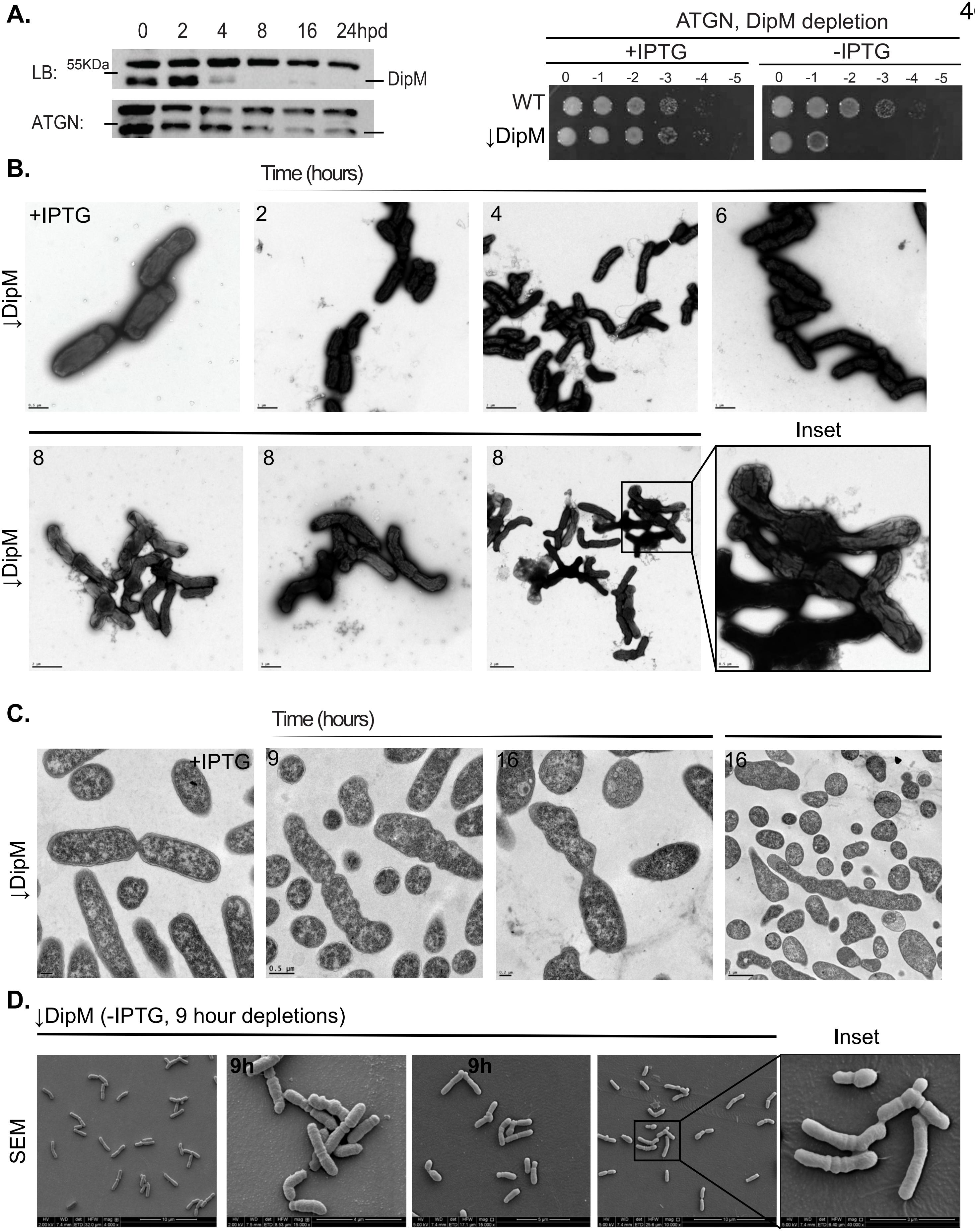

Supplementary Figure 7. Morphological defects increase over time during DipM depletion. (A, left) Western blots reveal the time course of DipM depletion in LB and ATGN. (A, right) Viability spot assays of WT and DipM depletion strain in ATGN in the presence and or absence of IPTG. (B) Whole cell transmission electron microscopy of replete (+IPTG) and DipM depleted cells at 2, 4, 6, and 8 hours of depletion in LB. Note, scale bars are not uniform and are (moving left to right and top to bottom) 0.5 µm, 1 µm, 2 µm, 1 µm, 2 µm, 1 µm, 2 µm, and 0.5 µm. (C) Thin section transmission electron microscopy of replete (+IPTG) and DipM depleted cells after 9 hours of depletion in LB. Scale bars are (moving left to right) 0.2 µm, 0.5 µm, 0.2 µm, 1 µm. (D) Scanning electron microscopy of DipM depleted cells after 9 hours of depletion in LB. Scale bars are 10 µm, 4 µm, 5 µm, 10 µm, and 3 µm.

3 Supplementary Video Captions

Supplementary Video 1. Growth of WT *A. tumefaciens c*ells on agarose pads. DIC image series of WT growing on a 1.25% LB agarose pad. Images were acquired every 10 minutes and the movie is played at 8 frames per second for a total of 43 frames. Scale bar = 2 µm.
Supplementary Video 2. Growth of RgsM depletion strain on agarose pads. DIC image series of the DipM depletion in the absence of the inducer IPTG and growing on a 1.25% ATGN agarose pad. Cells were pre-depleted in liquid 6 hours before starting the time-lapse. Images were acquired every 10 minutes and the movie is played at 8 frames per second for a total of 110 frames. Scale bar = 2 µm.
Supplementary Video 3. Growth of hypercurved Δ*envC* cells on agarose pads. DIC image series of Δ*envC* growing on a 1.25% LB agarose pad. Images were acquired every 10 minutes and the movie is played at 8 frames per second for a total of 91 frames. Scale bar = 2 µm.
Supplementary Video 4. Growth of DipM depletion on agarose pads. DIC image series of the DipM depletion in the absence of the inducer IPTG and growing on a 1.25% LB agarose pad. Cells were pre-depleted in liquid 6 hours before starting the time-lapse. Images were acquired every 5 minutes and the movie is played at 8 frames per second for a total of 77 frames. Scale bar = 2 µm.
Supplementary Video 5. Growth of hypercurved Δ*amiC* cells on agarose pads. DIC image series of Δ*amiC* growing on a 1.25% LB agarose pad. Images were acquired every 5 minutes and the movie is played at 8 frames per second for a total of 121 frames. Scale bar = 2 µm.
Supplementary Video 6. Growth of hypercurved Δ*envC*Δ*amiC* cells on agarose pads. DIC image series of Δ*envC*Δ*amiC* growing on a 1.25% LB agarose pad. Images were acquired every 5 minutes and the movie is played at 8 frames per second for a total of 96 frames. Scale bar = 2 µm.
Supplementary Video 7. Growth of Δ*amiC* cells during DipM depletion on agarose pads. DIC image series of the Δ*amiC* DipM depletion strain in the absence of the inducer IPTG and growing on a 1.25% LB agarose pad. Cells were pre-depleted in liquid 6 hours before starting the time-lapse. Images were acquired every 10 minutes and the movie is played at 8 frames per second for a total of 109 frames. Scale bar = 2 µm.
Supplementary Video 8. Growth of Δ*envC* cells during DipM depletion on agarose pads. DIC image series of the Δ*envC* DipM depletion strain in the absence of the inducer IPTG and growing on a 1.25% LB agarose pad. Cells were pre-depleted in liquid 6 hours before starting the time-lapse. Images were acquired every 10 minutes and the movie is played at 8 frames per second for a total of 102 frames. Scale bar = 2 µm.

4 Supplementary References

Choi K-H, Gaynor JB, White KG, Lopez C, Bosio CM, Karkhoff-Schweizer RR, Schweizer HP. 2005. A Tn7-based broad-range bacterial cloning and expression system. Nat Methods 2:443.

Figueroa-Cuilan W, Daniel JJ, Howell M, Sulaiman A, Brown PJB. 2016. Mini-Tn7 insertion in an artificial attTn7 site enables depletion of the essential master regulator ctrA in the phytopathogen *Agrobacterium tumefaciens*. Appl Environ Microbiol 82.

Figueroa-Cuilan W, Howell M, Richards C, Randich A, Yadav A, Cava F, Brown P. 2020. Induction of AmpC-mediated β-lactam resistance requires a single lytic transglycosylase in Agrobacterium tumefaciens. bioRxiv 1–52.

Khan SR, Gaines J, Roop RM, Farrand SK. 2008. Broad-host-range expression vectors with tightly regulated promoters and their use to examine the influence of TraR and TraM expression on Ti plasmid quorum sensing. Appl Environ Microbiol 74:5053–5062.

Simon R, Priefer U, Pühler A. 1983. A Broad Host Range Mobilization System for In Vivo Genetic Engineering: Transposon Mutagenesis in Gram Negative Bacteria. Bio/Technology 1:784–791.
